# Transformation of an early-established motor circuit during maturation in zebrafish

**DOI:** 10.1016/j.celrep.2022.110654

**Published:** 2022-04-12

**Authors:** Irene Pallucchi, Maria Bertuzzi, Jennifer Carlisle Michel, Adam C. Miller, Abdeljabbar El Manira

**Affiliations:** 1 Department of Neuroscience, Karolinska Institutet, 171 77 Stockholm, Sweden; 2 Institute of Neuroscience, University of Oregon, Eugene 97403, USA; 3 Lead contact

## Abstract

Locomotion is mediated by spinal circuits that generate movements with a precise coordination and vigor. The assembly of these circuits is defined early during development; however, whether their organization and function remain invariant throughout development is unclear. Here, we show that the first established fast circuit between two dorsally located V2a interneuron types and the four primary motoneurons undergoes major transformation in adult zebrafish compared with what was reported in larvae. There is a loss of existing connections and establishment of new connections combined with alterations in the mode, plasticity, and strength of synaptic transmission. In addition, we show that this circuit no longer serves as a swim rhythm generator, but instead its components become embedded within the spinal escape circuit and control propulsion following the initial escape turn. Our results thus reveal significant changes in the organization and function of a motor circuit as animals develop toward adulthood.

## In brief

Pallucchi et al. reveal that an early-established motor circuit in zebrafish undergoes a profound reorganization during maturation toward adulthood. There is a loss of existing connections and establishment of new connections combined with a change in function to suit new behavioral demands.

## Graphical Abstract

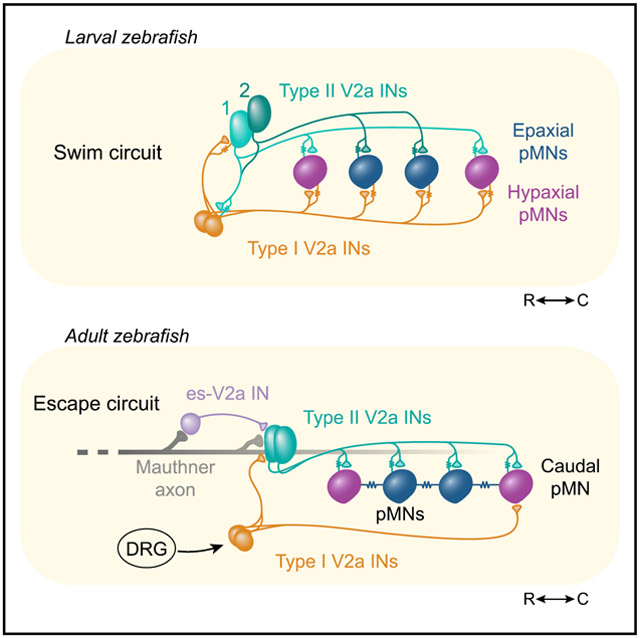

## INTRODUCTION

The execution of movements relies on a temporally precise activation of motor neurons to realize the planned actions (Grillner, 2003; Grillner and El Manira, 2020; Hooper and Büschges, 2017; Orlovsky et al., 1999; Wyart, 2018). In the spinal cord, functional circuits produce movements with precise timing, duration, and amplitude to adjust to changes in the environment (Arber, 2017; Brownstone and Bui, 2010; Büschges et al., 2011; Fetcho and McLean, 2010; Goulding, 2009; Grillner and Jessell, 2009; Hayashi et al., 2018; Kiehn, 2016; Roberts et al., 2010). The assembly of these circuits is defined early during development through processes that specify the constituent neurons’ identity and connectivity (Blankenship and Feller, 2010; Drapeau et al., 2002; Goulding and Pfaff, 2005; Ladle et al., 2007; Meng and Heckscher, 2021; Saint-Amant and Drapeau, 2000; Wan et al., 2019). Two recent studies in *C. elegans* and *Drosophila* have revealed that, during maturation, the decision-making circuitry is maintained unchanged, whereas sensory and motor pathways show age-dependent structural changes (Lee and Doe, 2021; Witvliet et al., 2021). In vertebrates, we have a broad understanding of the molecular mechanisms underlying circuit assembly and how the motor output and modulation of these circuits are reconfigured during development (Combes et al., 2004; Hachoumi and Sillar, 2020; Husch et al., 2015; Landmesser, 2018; Marder and Rehm, 2005; Saint-Amant and Drapeau, 2000). However, the nature of the changes in motor circuit connectivity during maturation has remained an ongoing challenge. Addressing this issue requires access to the same circuit components at the level of single neurons and synapses at different developmental stages.

The zebrafish is one of the few vertebrate model systems that affords the comparison of motor circuit organization and function at both larval and adult stages (Ahrens et al., 2012; Arrenberg et al., 2009; Berg et al., 2018; Buss and Drapeau, 2001; Chow et al., 2020; Dunn et al., 2016; Fetcho et al., 2008; Knafo and Wyart, 2018; McLean et al., 2008; Satou et al., 2013). Analysis of the spinal circuits driving locomotion has revealed changes in their organization and functional connectivity, which occur at the time when fish acquire adult features (Budick and O’Malley, 2000; Buss and Drapeau, 2001, 2002; Devoto et al., 2006; Gabriel et al., 2008; Hale, 2014; Jackson and Ingham, 2013; Kyriakatos et al., 2011; Masino and Fetcho, 2005; Müller et al., 2000; Picton et al., 2021; Saint-Amant and Drapeau, 1998; van Raamsdonk et al., 1982). In larvae, episodic swimming movements are mainly driven by fast circuits activating fast muscles (Kimura et al., 2006; McLean et al., 2007, 2008; McLean and Fetcho, 2009; Menelaou and McLean, 2012; Myers, 1985; Pujala and Koyama, 2019; Svara et al., 2018). In adults, the swimming network comprises three speed circuit modules that are activated sequentially with increased speed and vigor of movements (Ausborn et al., 2012; Gabriel et al., 2011; Song et al., 2018, 2020).

The first established motor circuit in zebrafish includes the two types of early-born V2a interneurons (INs; type I and type II) and primary motoneurons (pMNs) (Eisen et al., 1986; Kimura et al., 2006; McLean and Fetcho, 2009; McLean et al., 2008; Menelaou and McLean, 2012, 2019; Myers, 1985; Myers et al., 1986; Svara et al., 2018; Westerfield et al., 1986). Recent work in larval zebrafish has shown a distinct pattern of synaptic connections within this circuit (Bagnall and McLean, 2014; McLean et al., 2007; McLean and Fetcho, 2009; Menelaou and McLean, 2019). The two V2a IN types form a reciprocal excitatory circuit acting as a swim rhythm generator and connect differentially to pMNs. Type I V2a INs are connected broadly to all pMNs, whereas type II V2a INs seem to be selectively connected to the two pMNs innervating either epaxial or hypaxial fast muscles (Menelaou and McLean, 2019). Whether the neuronal components of this early established circuit, their connectivity and role in swimming are retained in adult stages remains unclear.

In this study, we reveal in zebrafish that a motor circuit, established and functional early in an animal’s life, reorganizes its connections and function by adulthood. We first establish that the two V2a IN types and pMNs in adult zebrafish can be individually identified and retain their larval features. Second, using sequential *ex vivo* paired recordings and behavioral analysis *in vivo*, we show that major changes occur in connectivity within this circuit and its function compared with early life. Type I V2a INs connect exclusively to the so-called caudal pMNs, innervating the ventralmost quadrant of the hypaxial muscles, through facilitating chemical synapses. Type II V2a INs, however, connect broadly to all pMNs over several segments, irrespective of the muscle quadrant they innervate, via non-facilitating mixed electrical and chemical synapses. Furthermore, we show that this circuit no longer serves as a rhythm generator for swimming, but instead its components become embedded within the spinal escape circuit. Thus, our results show that the first established fast circuit in zebrafish undergoes changes in organization and function during development toward adulthood.

## RESULTS

### The neural components of the early-established fast circuit in adult zebrafish

**Figure 1.**
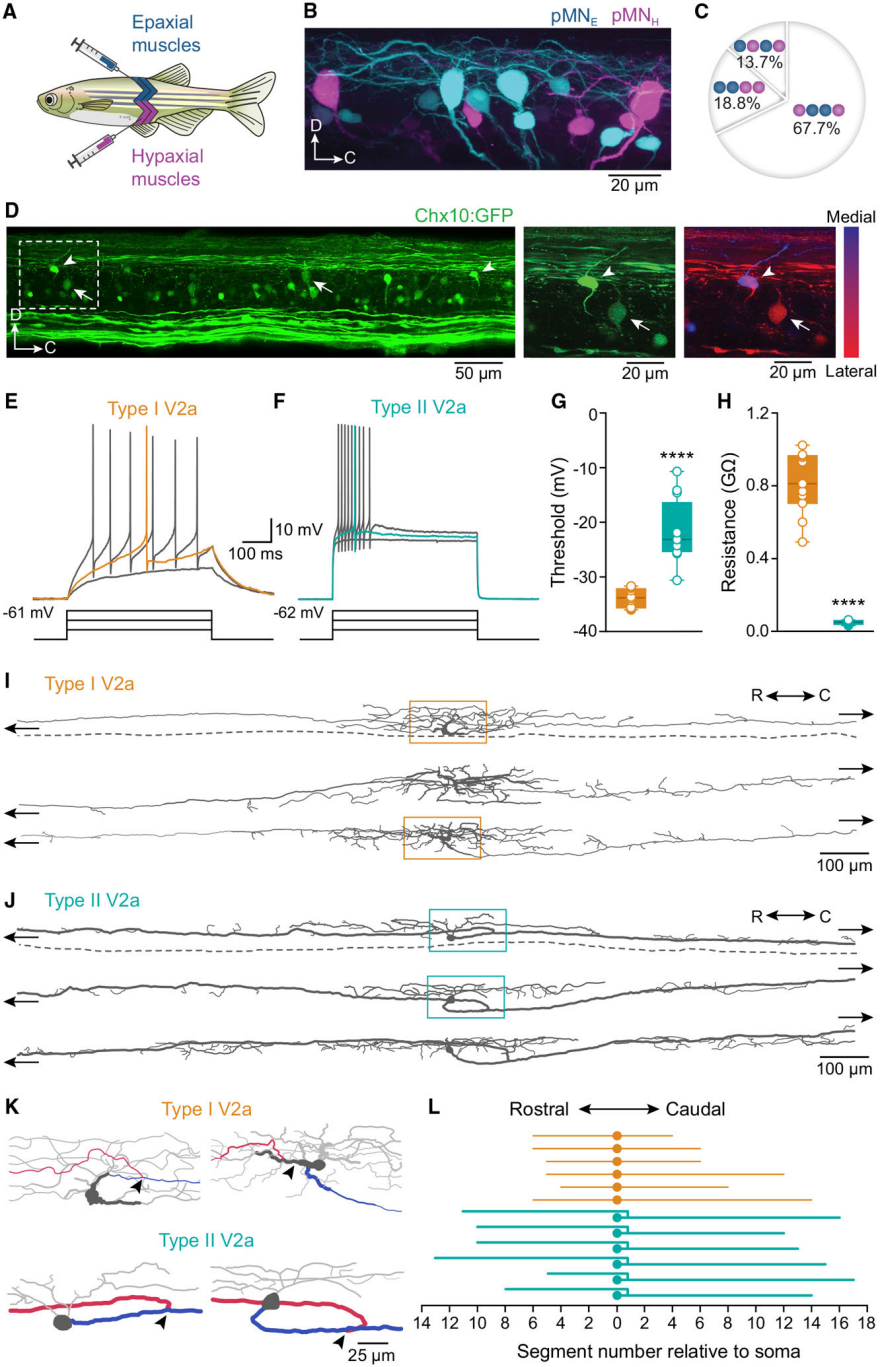
Components of the early-established fast circuit in adult zebrafish (A) Dextran dye injections in epaxial and hypaxial muscles in intact zebrafish. (B) Lateral view of one hemisegment of the spinal cord showing the soma position of epaxial (pMN_E_, cyan) and hypaxial (pMN_H_, magenta) pMNs. (C) Variability of rostrocaudal position occupied by epaxial (blue circles) and hypaxial (magenta circles) pMNs. The position of the caudal pMN innervating ventral hypaxial muscles was invariable (n = 85 segments, 11 animals). (D) Left: lateral view of two spinal segments showing the distribution of V2a INs. Middle: image of the part indicated by the dashed box showing a type I V2a IN with high GFP expression (arrowhead) and a type II V2 IN with low GFP expression (arrow). Right: depth analysis of the location of type I V2a (arrowhead) and type II V2a (arrow) INs along the mediolateral axis (9 mm orthogonal projection of the medial spinal cord). (E) A type I V2a IN displaying tonic firing in response to depolarizing current pulses. Orange trace shows threshold firing (n = 50 type I, 36 animals). (F) A type II V2a IN showing a strong adaptive firing in response to depolarizing current pulses. Green trace shows threshold firing (n = 52 type II, 44 animals). (G) Plot of types I and II V2a IN firing thresholds (****p ≤ 0.0001, two-tailed unpaired Student’s t test, n = 12 type I V2a INs; 9 animals, n = 12 type II V2a INs, 12 animals). (H) Plot of types I and II V2a IN input resistances (****p ≤ 0.0001, two-tailed unpaired Student’s t test, replicates as in [G]). (I) Morphologies of type I V2a INs. Top: dorsal view, with a dashed line indicating the midline. Middle and bottom: lateral views (n = 6 type I, 6 animals). (J) Morphologies of type II V2a INs. Top: dorsal view, with a dashed line indicating the midline. Middle and bottom: lateral views (n = 7 type II, 7 animals). (K) High-magnification views of the areas indicated by the boxes in (I) (top: type I V2a INs) and (J) (bottom, type II V2a INs). Arrowheads indicate the axon bifurcation point. The ascending axonal branch (>2 segments) is indicated in red and the descending axonal branch (>2 segments) in blue. (L) Length of the axonal projections of type I (orange) and type II (green) V2a INs. See also Figures S1 and S2.

To determine the organization and innervation pattern of pMNs in adult zebrafish and compare them with previous reports in larval and adult animals (Eisen et al., 1986, 1990; Menelaou and McLean, 2012; Myers, 1985; Myers et al., 1986; Westerfield et al., 1986), we injected retrograde dyes into the fast axial musculature (Figure 1A). Several fast MNs were labeled, including four pMNs per hemisegment (Figure 1B). To determine which of these pMNs innervate hypaxial or epaxial fast muscles, we injected two different retrograde dyes into these muscles. We consistently found two hypaxial and two epaxial pMNs per hemisegment (Figures 1B, 1C, and S1A), similar to what has been reported in larvae (Eisen et al., 1986, 1990; Menelaou and McLean, 2012; Myers, 1985). One of the hypaxial pMNs always occupied the caudalmost position in the segment, whereas the other hypaxial and the two epaxial pMNs had variable soma positions (Figure 1C, *n* = 85 segments, 11 animals). This caudalmost hypaxial pMN innervated the ventral quadrant of ipsilateral fast axial musculature (Figure S1B; *n* = 57 segments, 5 animals), consistent with results from previous studies in larva and adult (Eisen et al., 1986; Menelaou and McLean, 2012; Myers et al., 1986; Westerfield et al., 1986).

In larval zebrafish (4–5 days post-fertilization [dpf]; 3–4 mm long), two early-born V2a IN types (I and II) are dorsally located and were identified based on their level of expression of the transcription factor *Chx10* (inferred by the GFP level), input resistance, and firing pattern (Menelaou and McLean, 2019). Type I V2a INs have a high *Chx10* expression and high input resistances, fire regularly with little adaptation, and show a unidirectional axonal projection (Menelaou and McLean, 2019). Type II V2a INs have a lower *Chx10* expression, are relatively more ventral, have low input resistances, display a strong firing adaptation, and have bidirectional axonal projections (Menelaou and McLean, 2019). To determine whether these two V2a IN types could still be identified in adult zebrafish, we focused on the most dorsal neurons in the transgenic (Tg)(*Chx10*:GFP) line. On the basis of the level of *Chx10* expression, we could identify two V2a IN types located in the dorsal aspect of the spinal cord. There were one or two neurons of each type per hemisegment (Figure 1D). One type of V2a INs had the highest level of *Chx10* expression and was located most dorsally and medially among all the V2a INs and could correspond to larval type I (Figure 1D; arrowhead). The other V2a IN type showed low levels of *Chx10* expression, seen as weaker GFP intensity, and was located more ventrally at the level of pMNs. This type could correspond to larval type II V2a INs (Figure 1D; arrow).

To determine whether these presumptive type I and II V2a INs display similar physiological and morphological features to those in larvae, we performed whole-cell patch-clamp recordings using an *ex vivo* preparation of the adult zebrafish spinal cord (Ausborn et al., 2012; Gabriel et al., 2008; Kyriakatos et al., 2011). The *Chx10*-high, dorsomedial V2a IN showed regular firing with little adaptation in response to depolarizing current injections (Figure 1E; *n* = 50 neurons, 36 animals), had a low firing threshold and high input resistance (Figures 1G and 1H; *n* = 12 neurons, 9 animals), strongly resembling the type I V2a INs reported in larvae. In contrast, the *Chx10*-low, ventrolateral V2a INs fired at a high frequency with a strong adaptation (Figure 1F; *n* = 52 neurons, 44 animals) and had a high firing threshold and a low input resistance (Figures 1G and 1H; *n* = 12 neurons, 12 animals), similar to the larval type II V2a INs.

Morphological analysis revealed that *Chx10*-high V2a INs had small-diameter axons with visible collaterals (Figures 1I and S1D; *n* = 6 neurons, 6 animals), while *Chx10*-low V2a INs had large-diameter axons with many collaterals both in the rostral and caudal portions (Figures 1J and S1D; *n* = 7 neurons, 7 animals). A close analysis of the soma and axonal branching showed that *Chx10*-high V2a INs had a small soma (Figure S1C) with extensive dendritic arborization extending dorsally and a thick initial axon segment that bifurcated in the segment where their soma is located (Figure 1K, top). *Chx10*-low V2a INs, on the other hand, had a large soma (Figure S1C) and a less extensive dendritic arborization that extended along the rostrocaudal axis (Figure 1K, bottom). This type had a main descending projection with large diameter, which gave rise to an ascending collateral (Figure 1K, bottom). Both V2a IN types had axonal projections that extended over several segments in both directions, but only *Chx10*-low V2a INs (type II) extended their axons along the full length of the spinal cord to reach the brainstem and the caudal end of the cord (Figures 1L and S1E).

Finally, to confirm that types I and II V2a INs described in larvae correspond to those we have identified in adult zebrafish, the two V2a types were ablated over four to nine segments in larvae (4–5 dpf, *n* = 6 animals; Figures S2A-S2C), and these animals were examined when they had developed to adult stages. Whereas V2a INs with high and low *Chx10* expression could be identified in the dorsal aspect of the adult spinal cord in non-ablated segments, they were completely absent in the segments where they were ablated at larval stages (Figure S2D). These results, together with our analysis of molecular, physiological, and morphological properties, and a comparison with larval identities, support the notion that the *Chx10*-high and the *Chx10*-low V2a INs represent type I and type II V2a INs, respectively.

### Synaptic connectivity of V2a IN types and pMNs

**Figure 2.**
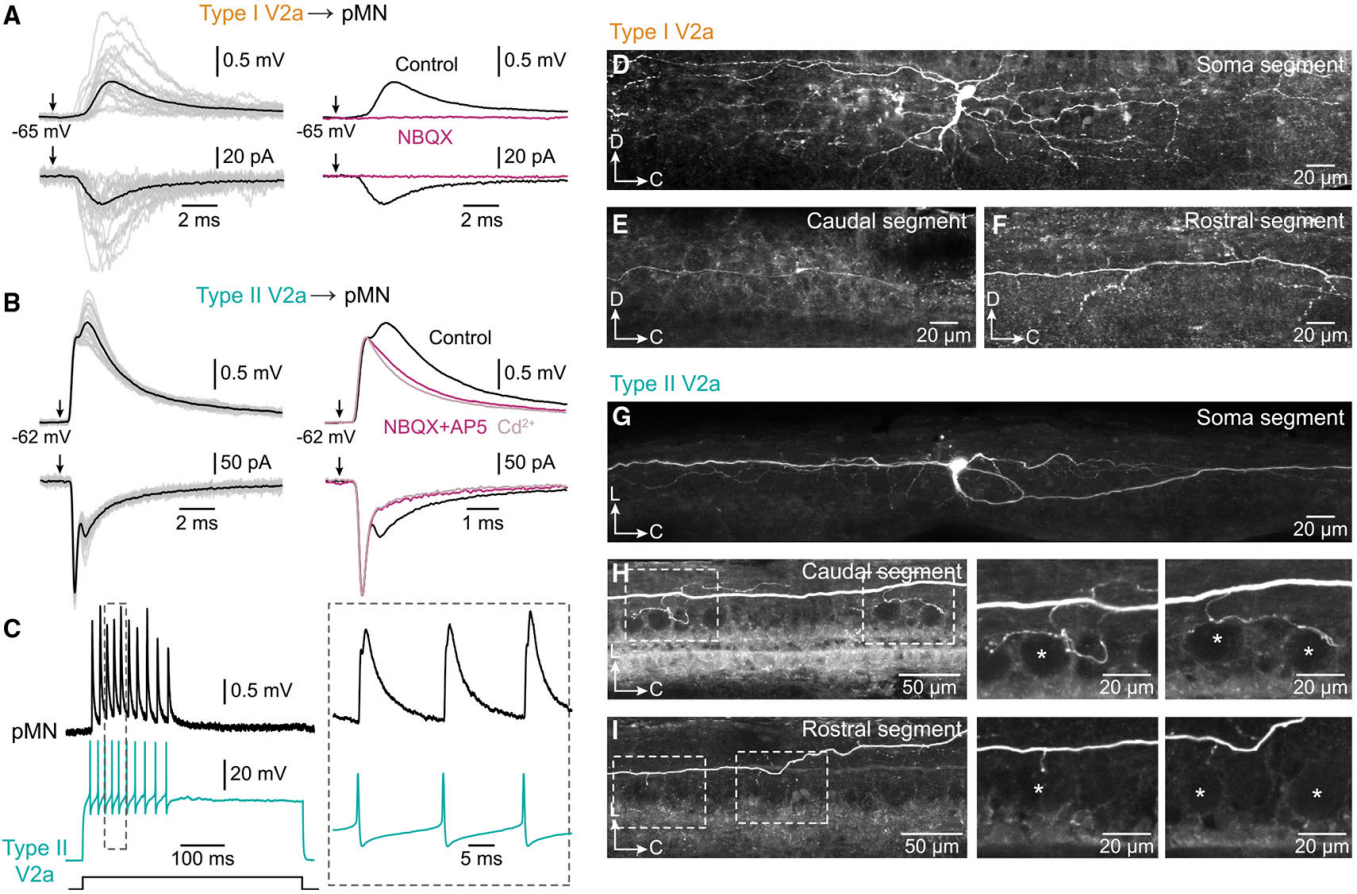
Synaptic connections between type I or type II V2a INs and pMNs (A) Type I V2a IN stimulation elicited a chemical EPSP/C in a pMN (left), that was abolished by NBQX (right). Gray traces, 20 individual sweeps; black and magenta traces, averages of 50 sweeps. Arrows indicate V2a IN action potentials (n = 32 type I, 18 animals). (B) Type II V2a IN stimulation elicited a mixed chemical and electrical EPSP/C in a pMN (left, n = 85 type II, 42 animals). The chemical component was abolished by NBQX and AP5, leaving only an electrical component that remained also in presence of cadmium (Cd^2+^, right, n = 3 type II, 3 animals). Gray traces, 20 individual sweeps, black magenta and pink traces, averages of 80 sweeps. Arrows indicate V2a IN action potentials. (C) Repetitive action potentials in a type II V2a IN elicited large mixed EPSPs in a pMN. (D) Lateral view of a neurobiotin-filled type I V2a IN (n = 12 type I, 12 animals). (E) Caudal axonal projection of a type I V2a IN. (F) Rostral axonal projection of a type I V2a IN. (G) Dorsal view of a neurobiotin-filled type II V2a IN (n = 14 type II, 14 animals). (H) Close proximities between collaterals, arising from a type II V2a IN descending axon, and pMN somata (indicated by asterisks). Right: high magnification of the parts indicated by dashed boxes at the left. (I) Short collaterals arising from a type II V2a IN ascending axon running close to pMN somata (indicated by asterisks).

To determine whether the two types of V2a INs connect to pMNs in adult zebrafish as in larvae, we performed dual whole-cell patch-clamp recordings. Type I V2a INs were connected to pMNs exclusively via chemical synapses, and the resulting slow excitatory postsynaptic potentials/currents (EPSP/Cs) were eliminated by the AMPA receptor antagonist NBQX (Figure 2A; *n* = 32 type I, 18 animals). Type II V2a INs were also connected to pMNs, but via mixed electrical and chemical synapses (Figure 2B, left; *n* = 85 type II, 42 animals). The chemical component of the EPSP/Cs (cEPSP/C) was blocked by the glutamate receptor antagonists NBQX and AP5, while the remaining fast electrical component (eEPSP/C) persisted also in cadmium (Figure 2B, right; *n* = 3 type II, 3 animals). Furthermore, single action potentials in type II V2a INs elicited large and reliable EPSPs in pMNs even during high-frequency firing (Figure 2C).

Morphological analysis shows that the axons of type I V2a INs always ran more lateral than the pMN somata, had a variable dorso-ventral position, and gave rise to sparse axon collaterals with no detectable close contacts with pMN somata (Figures 2D-2F; *n* = 12 type I, 12 animals). In contrast, type II V2a IN axonal projections were highly consistent, projecting more medially with many collaterals wrapping pMN somata (Figures 2G-2I; *n* = 14 tpye II, 14 animals).

Together, these results show that pMNs and the types I and II V2a INs in adult zebrafish conserve several characteristic features similar to those in larvae. This neural network provides a system to examine whether the selective targeting observed in larval stages is maintained as the nervous system matures.

### Selectivity and short-term plasticity of type I V2a IN-pMN synapses

**Figure 3.**
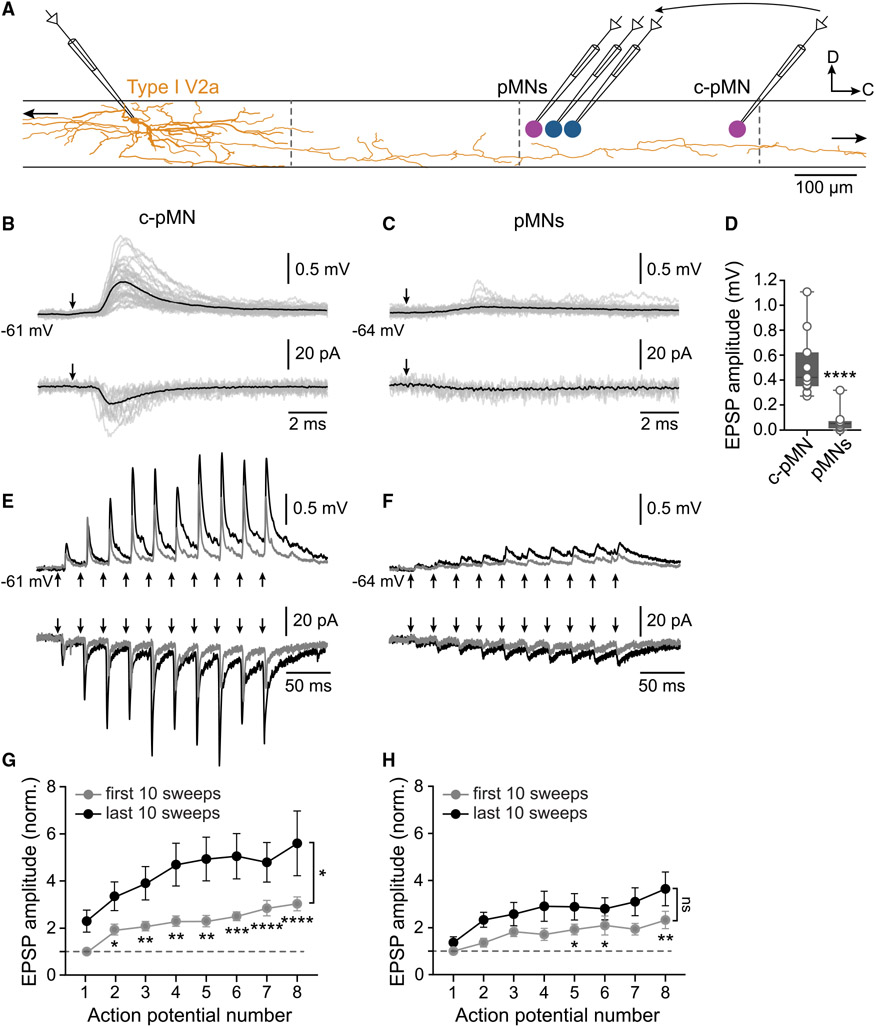
Selective synaptic connections between type I V2a INs and pMNs (A) Experimental setup for sequential paired recordings showing a type I V2a IN reconstruction (orange) and the four pMNs in a hemisegment (blue, epaxial; magenta, hypaxial). Dashed lines indicate spinal segment borders (n = 13 type I V2a INs, 13 animals). (B) Type I V2a IN stimulation elicited EPSP/Cs in the caudal most pMN in a hemisegment. Gray traces, 20 individual sweeps; black trace, average of 100 sweeps. Arrows indicate V2a IN action potentials. (C) Type I V2a IN stimulation did not elicit any EPSP/Cs in the three other pMNs. Gray traces, 20 individual sweeps; black trace, average of 100 sweeps. Arrows indicate V2a IN action potentials. (D) Box and whiskers plot of the EPSP amplitude in pMNs (****p ≥ 0.0001, non-parametric Mann-Whitney test, type I V2a IN to c-pMNs n = 11 pairs, 7 animals, type I V2a IN to other pMNs n = 10 pairs, 8 animals). (E) Example traces showing short-term facilitation of EPSP/Cs elicited in a caudal pMN in response to 40-Hz stimulation of a type I V2a IN. Gray traces, average of sweeps 1–10 (0–20 s); black traces, averages of sweeps 71–80 (140–160 s). Arrows indicate V2a IN action potentials. (F) Example traces showing EPSP/Cs elicited in the other three pMNs in response to 40-Hz stimulation of a type I V2a IN. (G) Amplitude of EPSPs elicited in caudal pMNs by 40-Hz stimulation of type I V2a INs (n = 8 pairs, 7 animals). (H) Amplitude of EPSPs elicited in the other pMNs by 40-Hz stimulation of type I V2a INs (n = 8 pairs, 8 animals; in [G] and [H] data are presented as mean ± SEM, *p ≤ 0.05, **p ≤ 0.01, ***p ≤ 0.001, ****p ≤ 0.0001; one-way repeated-measures ANOVA for the first 10 sweeps. Two-way repeated-measures ANOVA for the last 10 sweeps). See also Figure S3.

We next wanted to determine whether connectivity patterns were maintained into adulthood, first by examining if type I V2a INs make uniform synaptic connections with all hypaxial and epaxial pMNs, as observed in larvae (Menelaou and McLean, 2019). For this, we sequentially sampled connections between a single V2a IN and the four pMNs (Figure 3A; *n* = 13 type I V2a INs, 13 animals). Type I V2a INs connected selectively with the caudalmost pMN (c-pMN) in each segment (Figures 3B and 3D; *n* = 11 pairs, 7 animals). This connection showed a strong time- and use-dependent facilitation during repeated high-frequency stimulation (Figures 3E and 3G; *n* = 8 pairs, 7 animals). By contrast, virtually no EPSPs or EPSCs were elicited in the other three pMNs by single action potentials in type I V2a INs (Figures 3C and 3D; *n* = 10 pairs, 8 animals). However, weak EPSP/Cs could be revealed in response to repeated high-frequency stimulation of this V2a IN type (Figures 3F and 3H; *n* = 8 pairs, 8 animals). On the other hand, type I V2a INs did not make synaptic contacts with any of the pMNs located in rostral segments (Figure S3; *n* = 5 c-pMN, *n* = 3 other pMNs, 6 animals). These results show that type I V2a INs make strong and selective facilitating chemical synapses primarily with c-pMNs.

### Widespread synaptic connections between type II V2a INs and pMNs

**Figure 4.**
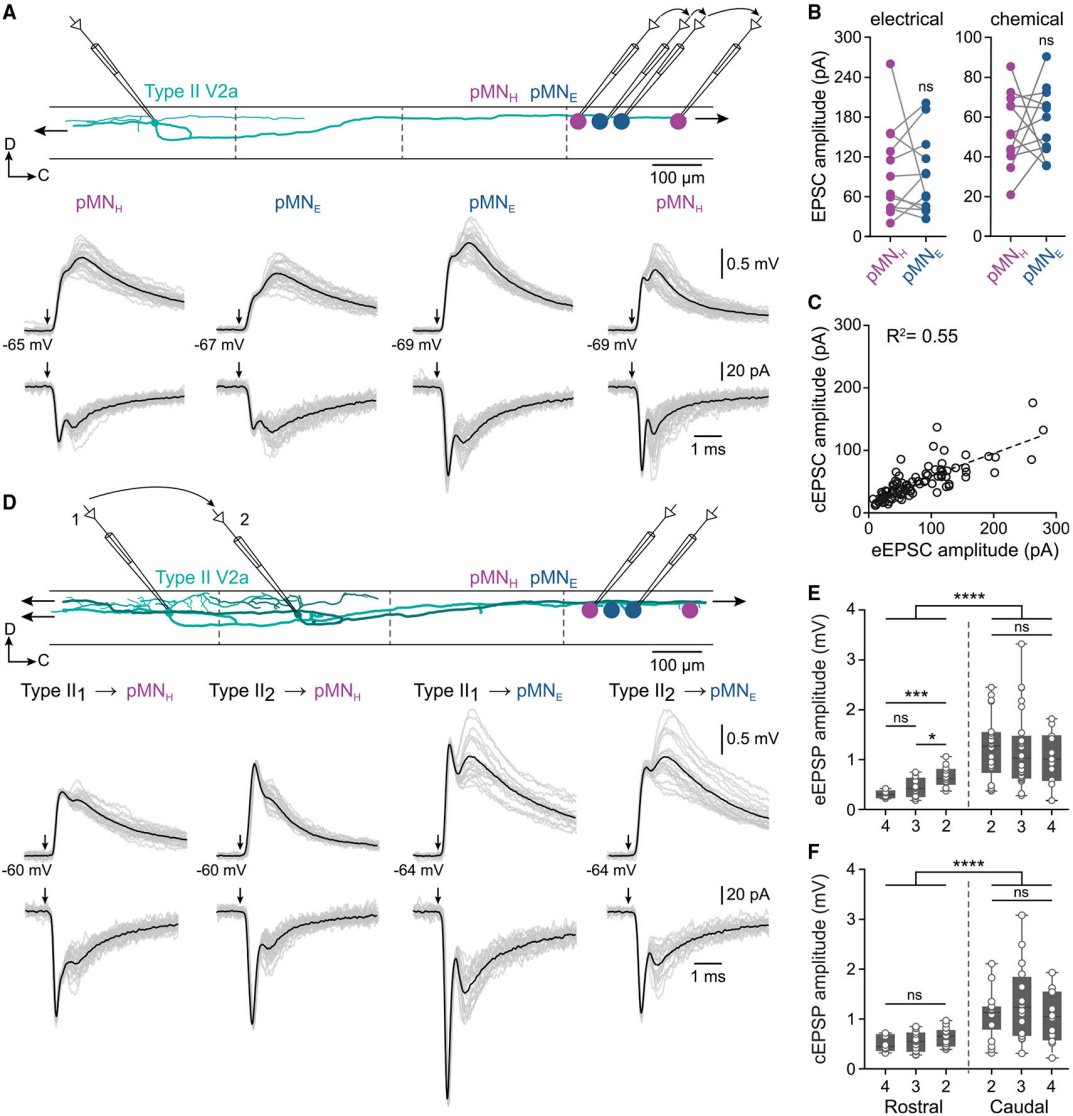
Broad synaptic connections between type II V2a INs and pMNs (A) Top: experimental setup of sequential paired recordings showing a reconstruction of a type II V2a IN (green) and epaxial (pMN_E_, blue) and hypaxial (pMN_H_, magenta) pMNs. Dashed lines indicate spinal segment borders. Bottom: example EPSP/Cs elicited in the four pMNs by stimulation of the same type II V2a IN. Gray traces, 20 individual sweeps; back traces, averages of 50 sweeps. Arrows indicate V2a IN action potentials (n = 29 V2a INs, 86 pairs, 26 animals). (B) Amplitude of chemical and electrical EPSCs elicited in pMNs by stimulation of the same type II V2a IN (ns, not significant; paired Student’s t test, n = 12 hypaxial and 12 epaxial pMNs). (C) Correlation between cEPSC and eEPSC amplitude (R^2^ = 0.55, p < 0.0001, n = 88). (D) Top: experimental setup of sequential paired recordings with two reconstructed type II V2a INs (green). Two type II V2a INs were recorded sequentially while holding either a pMN_E_ or a pMN_H_. Bottom: example traces of EPSP/Cs elicited in the same pMN_H_ or pMN_E_ by stimulation of two different type II V2a INs (n = 6 pMNs, 6 animals). (E and F) Plot of eEPSP (E) or cEPSP (F) amplitude in pMNs recorded in caudal or rostral segments relative to the stimulated type II V2a IN (*p ≤ 0.05, ***p ≤ 0.001, ****p ≤ 0.0001; ns, not significant; one-way ANOVA with Tukey’s multiple comparisons, unpaired Student’s t test for comparison of EPSPs in pMNs in rostral and caudal segments; n = 35 pMNs rostral, n = 58 pMNs caudal, 34 animals). See also Figure S4.

We next examined whether the selective connectivity of larval type II V2a INs with pMNs innervating hypaxial/epaxial fast muscle is preserved in adult fish. We sequentially sampled connections between a single type II V2a IN and all four pMNs located in a segment caudal to the IN (Figure 4A; *n* = 29 V2a INs, 26 animals). Hypaxial and epaxial pMNs (pMN_H_ and pMN_E_) were pre-labeled by retrograde dye injections into their respective muscles prior to every experiment. Action potentials in a single type II V2a IN elicited strong and reliable EPSP/Cs in both the hypaxial and epaxial pMNs (Figure 4A, *n* = 86 pairs; Figure 4B, *n* = 12 hypaxial and 12 epaxial pMNs). Synapses between type II V2a IN and pMNs were always stronger compared with those between type I V2a INs and c-pMNs (Figure S4C; n = 13 type I, 63 type II V2a INs), but they did not show time-oruse-dependent facilitation during repeated high-frequency stimulation (Figures S4A and S4B; *n* = 6 pairs, 6 animals). There was a strong correlation between the amplitude of the eEPSCs and cEPSCs (Figure 4C; *n* = 88 pairs), suggesting that the strength of the electrical and chemical components co-varies in a synapse-dependent manner.

To examine whether different type II V2a INs converge on a single hypaxial or epaxial pMN, we sequentially sampled the connections of type II V2a INs in distinct segments with pMNs in a single caudal segment (Figure 4D). In all experiments, both hypaxial and epaxial pMNs received converging mixed electrical and chemical EPSP/Cs from all the sampled type II V2a INs (Figure 4D; *n* = 6 pMNs, 6 animals).

The selectivity of synaptic connections was also tested between type II V2a INs and pMNs located in rostral segments (Figure S4D). In all cases, single action potentials in type II V2a INs elicited EPSP/Cs in both hypaxial and epaxial pMNs (Figures S4D-S4F; *n* = 19 V2a INs, 18 animals). However, the amplitude of both the electrical and chemical EPSP/Cs was significantly smaller in these pMNs compared with those located in caudal segments (Figures 4E and 4F; *n* = 35 pMNs rostral, *n* = 58 pMNs caudal, 34 animals).

Together, these results show that type II V2a INs connect broadly to both hypaxial and epaxial pMNs using non-facilitating mixed electrical and chemical synapses. In addition, we show that this V2a IN type provides both divergent and convergent synaptic inputs that are uniformly distributed onto all pMNs regardless of their muscle innervation pattern and segmental distance.

### Gap junction composition at type II V2a IN-pMN synapses

**Figure 5.**
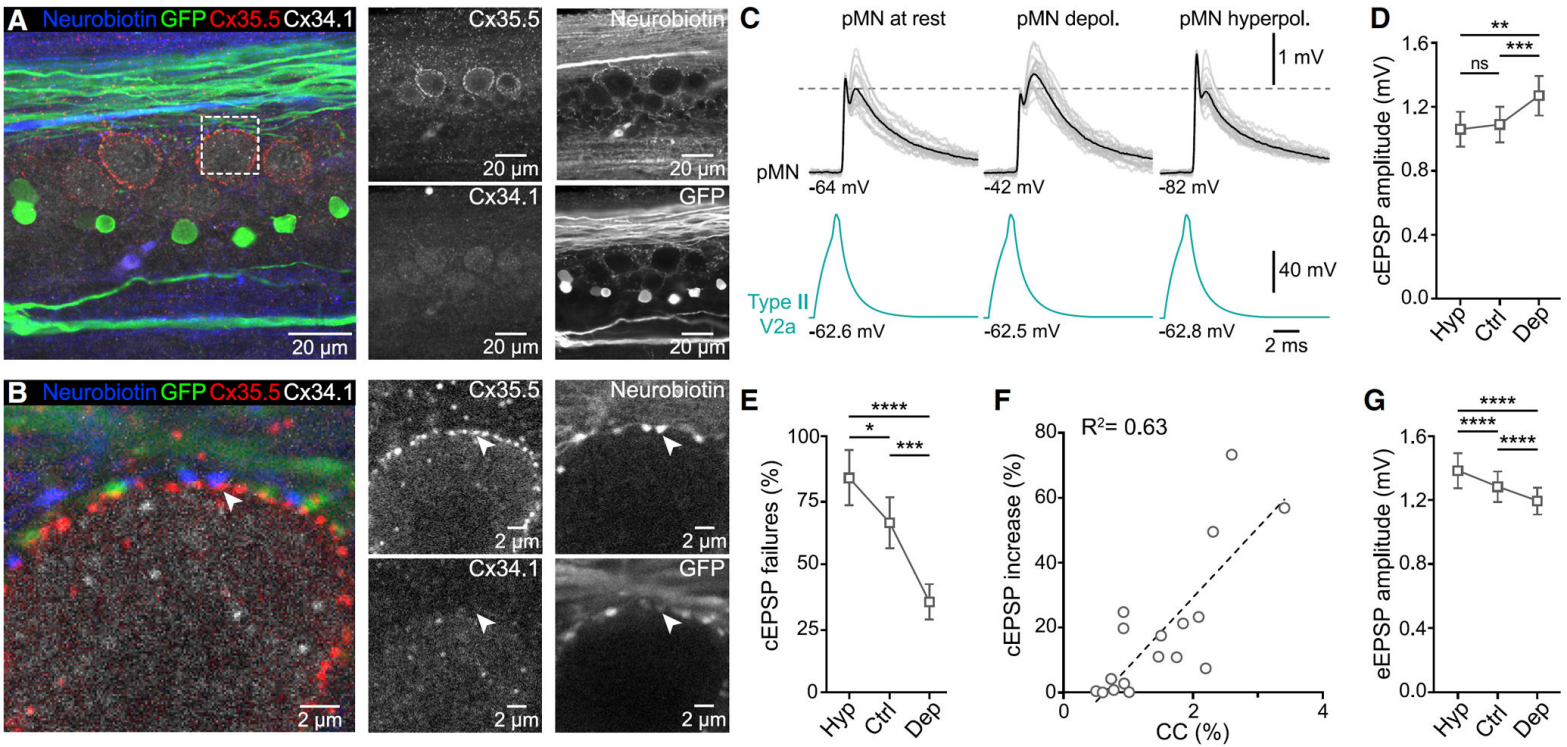
pMNs influence type II V2a IN synaptic transmission retrogradely via gap junctions (A) Axon collaterals from neurobiotin-filled type II V2a IN wrap-around pMN somata, which express connexin 35.5 (Cx35.5), but not Cx34.1 (n = 6 type II, 6 animals). (B) High magnification of the area indicated by the dashed box in (A) at a single focal plan showing close contacts between type II V2a IN collaterals and a pMN soma. The presynaptic collaterals do not express Cx35.5, which seems to be restricted to the postsynaptic pMN. (C) Representative recordings from a type II V2a IN and a pMN showing that the EPSP amplitude is strongly influenced by the pMN membrane potential. Gray traces, 20 individual sweeps; black and green traces, averages of 50 sweeps. (D) Change in the cEPSP amplitude as a function of the pMN membrane potential (data are presented as mean ± SEM, *p ≤ 0.05, **p ≤ 0.01, ***p ≤ 0.001, ****p ≤ 0.0001, one-way repeated-measures ANOVA corrected with Tukey’s multiple comparisons test, n = 44 pairs, 25 animals). (E) Changes in the cEPSP failure rate as a function of the pMN membrane potential (statistical test and replicates as in [D]). (F) Correlation between cEPSP amplitude and coupling coefficient (R^2^ = 0.63, p < 0.001, n = 17 pairs, 14 animals). (G) Changes in the eEPSP amplitude as a function of the pMN membrane potential (statistical test and replicates as in [D]). See also Figures S5 and S6.

In the escape spinal circuit of larval zebrafish, gap junctions between Mauthner cell axons (M-axons) and commissural local (CoLo) INs were shown to be molecularly asymmetric with unique pre- and postsynaptic connexins. Connexin 35.5 is localized presynaptically, in the axonal compartment, while connexin 34.1 was found postsynaptically, within the somatodendritic compartment (Miller et al., 2017). To test if these connexins are present at gap junctions between type II V2a INs and pMNs, we used the same antibodies that were shown to be specific to each protein (Miller et al., 2017). This immunohistochemical analysis was performed in preparations with neurobiotin-filled type II V2a INs (Figure 5A; *n* = 6 type II, 6 animals). In several segments, collateral branches of the type II V2a INs wrapped the soma of pMNs, which were richly decorated with connexin 35.5 over the whole surface (Figures 5A, 5B, and S5A). The pMNs showed no apparent synaptic patterns of connexin 34.1 staining (Figures 5A and 5B), whereas other unidentified spinal cord neurons displayed clear punctate patterns of colocalizing Cx34.1/Cx35.5 on their cell bodies (Figure S5B). The axon collaterals of type II V2a INs formed varicosities that made close contacts with pMN somata and which were juxtaposed to connexin 35.5 puncta (Figures 5B and S5C). These results suggest that connexin 35.5 contributes to the gap junctions between type II V2a INs and pMNs.

The gap junctions between type II V2a INs and pMNs mediated electrical coupling as revealed using subthreshold hyperpolarizing current injections (Figure S5D; *n* = 48 pairs, 25 animals). The coupling coefficient was always larger from pMNs to V2a INs (Figures S5D and S5E) and decreased as a function of distance between the two neurons (Figure S5E). The difference in coupling coefficient in the two directions indicates that these electrical synapses are mediated by rectifying gap junctions.

This strong electrical coupling afforded by gap junctions could allow changes in pMN membrane potential to propagate retrogradely to presynaptic terminals of type II V2a INs and influence synaptic transmission (Song et al., 2016). To test this, we performed dual recordings from these neurons while holding the pMNs at hyperpolarized or depolarized membrane potentials (Figure 5C; *n* = 44 pairs, 25 animals). The amplitude of the cEPSP was increased by depolarization of the pMNs (Figures 5C and 5D), and failure rates were affected by both depolarization and hyperpolarization of the pMNs (Figure 5E). In addition, there was a significant correlation between the coupling coefficient between pMNs and V2a INs and the increase in the cEPSP amplitude by steady-state depolarization of pMNs (Figure 5F; *n* = 17 pairs, 14 animals). The eEPSP amplitude changed in the opposite direction compared with the cEPSP as it was increased by hyperpolarization and decreased by depolarization of pMNs (Figures 5C and 5G). These results show that gap junctions allow pMNs to exert a retrograde influence on the presynaptic type II V2a INs.

### Synchronization of pMNs firing through gap junctions

Primary MNs also displayed strong electrical coupling among themselves. Intracellular neurobiotin injections in a single pMN always resulted in dye-coupling of the other three pMNs located in the same segment (Figures S6A and S6B; *n* = 5 filled pMNs, 4 animals). The existence of electrical coupling was confirmed using dual whole-cell patch-clamp recordings, which showed bidirectional current flow between all four pMNs with a large coupling coefficient (Figures S6C and S6E; *n* = 14 pairs, 7 animals). When electrically coupled pMNs were simultaneously depolarized to firing threshold, their action potentials were highly synchronous (Figures S6F and S6H; *n* = 6 pairs, 4 animals). By contrast, secondary MNs (sMNs) innervating slow and intermediate muscles were weakly coupled (Figures S6D and S6E; *n* = 56 pairs, 35 animals) and did not display synchronized firing (Figures S6G and S6I; *n* = 3 pairs, 2 animals).

### Reorganization of type I and II V2a IN function and connectivity

**Figure 6.**
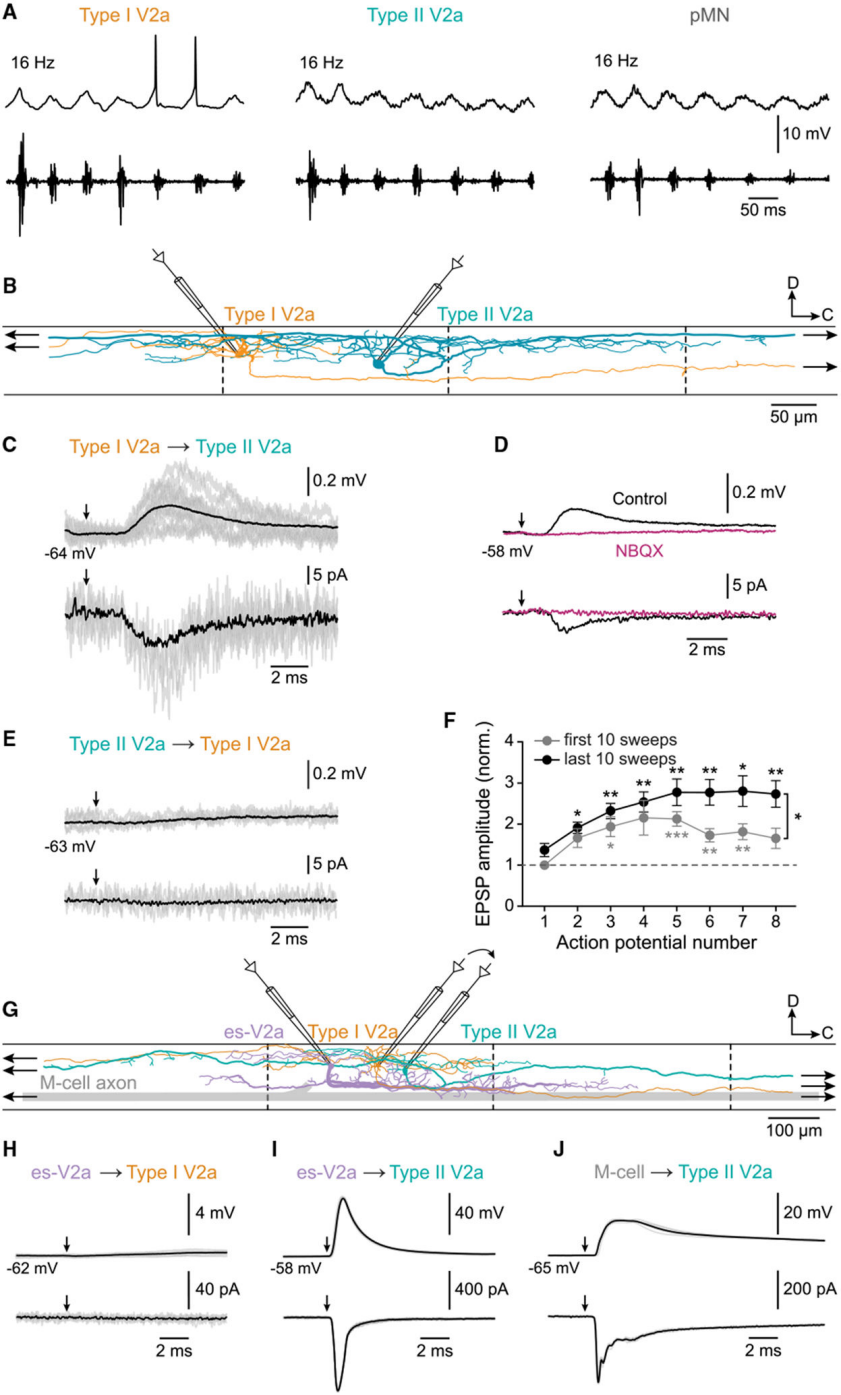
Activity and connectivity of types I and II V2a Ins (A) Types I and II V2a INs and pMNs were not recruited during fast swimming activity induced by optogenetic stimulation of hindbrain neurons. Top: intracellular recording; bottom: extracellular recording from a peripheral motor nerve (n = 3 each type, 5 animals). (B) Experimental setup of paired recordings showing the morphology of a type I and a type II V2a IN. Arrows indicate that their axons extend both rostrally and caudally. (C) Example traces of EPSP/Cs elicited in a type II V2a IN by stimulation of a type I V2a IN. Gray traces, 20 individual sweeps; black traces, averages of 150 sweeps. Arrows indicate V2a IN action potentials (n = 16 pairs). (D) Type I-induced EPSP/Cs in type II V2a INs was abolished by NBQX. Traces are averages of 150 sweeps (n = 5 pairs). (E) Lack of synaptic connection between a type II and a type I V2a INs (n = 16 pairs). (F) Short-term potentiation of EPSPs elicited in type II by stimulation of type I V2a INs (data are presented as mean ± SEM, *p ≤ 0.05, **p ≤ 0.01, ***p ≤ 0.001, one-way repeated-measures ANOVA for the first 10 sweeps. Two-way repeated-measures ANOVA for the last 10 sweeps, n = 16 pairs). (G) Experimental setup for sequential paired recordings between escape V2a INs (es-V2a, purple) and type I V2a IN (orange), and then type II V2a INs (green). (H) No EPSP/Cs were elicited in a type I V2a IN by stimulation of an es-V2a IN (n = 7 pairs, 6 animals). (I) Stimulation of an es-V2a IN elicited large EPSP/Cs in a type II V2a IN (n = 5 pairs, 5 animals). (J) Stimulation of a M-cell elicited compound EPSP/Cs in a type II V2a IN (n = 3 pairs, 3 animals). See also Figure S7.

In larvae, types I and II V2a INs form a positive feedback circuit via excitatory reciprocal connections that drive the activity of pMNs during fast swimming (Menelaou and McLean, 2019). Given the changes in morphology and connectivity of V2a INs and pMNs between larval and adult stages, we first examined whether these three neuronal components are recruited during fast swimming in adult zebrafish. Patch-clamp recordings were made from type I or type II V2a INs, or pMNs, while at the same time fictive swimming was induced by optogenetic stimulation of descending glutamatergic neurons in the brainstem. Both V2a IN types and pMNs displayed subthreshold membrane potential oscillations that were correlated with the swimming activity recorded in a peripheral motor nerve but were never reliably recruited at any frequency (Figure 6A; *n* = 3 each type, 5 animals). Type I V2a INs could occasionally fire action potentials that were not occurring reliably with every swimming cycle even at the highest frequencies (Figure 6A).

We next tested whether the two V2a IN types are reciprocally connected by use of dual patch-clamp recording (Figure 6B). Single action potential in type I V2a INs reliably elicited EPSP/Cs in type II V2a INs located either caudally or rostrally in the same segment (Figure 6C; *n* = 16 pairs). These EPSP/Cs were eliminated by the AMPA receptor antagonist NBQX (Figure 6D; *n* = 5 pairs), indicating that they were mediated only via chemical synapses. The amplitude of these chemical EPSP/Cs showed a strong time- and use-dependent facilitation during repetitive stimulation of type II V2a INs at high frequency (Figure 6F; *n* = 16 pairs). In contrast, stimulation of type II V2a INs did not induce any EPSP/Cs in type I V2a INs (Figure 6E; *n* = 16 pairs). These results show that types I and II V2a INs do not form a reciprocal circuit; rather, they are only connected unidirectionally via facilitating glutamatergic synapses.

Since the two V2a INs types do not contribute to the generation of swimming activity, we tested whether they become embedded within the circuit driving escape. This circuit in adult zebrafish comprises the descending command neurons, including M-cells (Lee and Eaton, 1991; Mauthner, 1859; Zottoli, 1977), and a recently identified spinal cholinergic V2a IN type (esV2a INs) (Guan et al., 2021). We therefore probed whether the two V2a IN types receive excitation from esV2a INs by using sequential dual recordings (Figures 6G, S7A, and S7B). Single action potentials in an esV2a IN did not produce any EPSP/Cs in type I V2a INs (Figure 6H; *n* = 7 pairs, 6 animals), but instead elicited large EPSP/Cs in type II V2a INs that often reached the threshold for action potentials (Figure 6I; *n* = 5 pairs, 5 animals). Type II V2a INs also received synaptic input from the M-cell consisting of large EPSP/Cs with multiple components (Figure 6J; *n* = 3 pairs, 3 animals), confirming that this V2a IN type is embedded within the escape circuit in adult zebrafish. To test whether these two V2a IN types are recruited during escape, we used the *ex vivo* brainstem-spinal cord preparation in which an escape motor pattern was induced by stimulation of the M-cell area and consisted of large motor bursts (Figure S7C [Song et al., 2015]). Type I V2a INs were not recruited during fictive escape and only displayed subthreshold depolarizations (Figure S7D; *n* = 5 type I, 3 animals). In contrast, type II V2a INs were always recruited during fictive escape and fired multiple action potentials (Figure S7E; *n* = 5 type II, 3 animals). Finally, to test whether these two V2a IN types receive sensory inputs, a dorsal root ganglion (DRG) was stimulated while whole-cell recordings were made from these interneurons (Figures S7F and S7G). Stimulation of DRG induced large compound EPSP/Cs in type I V2a (Figures S7H and S7J; *n* = 4 type I, 3 animals) but not in type II V2a INs (Figures S7I and S7J; *n* = 3 type II, 3 animals).

### Type II V2a INs are involved in controlling the propulsive phase during escape

**Figure 7.**
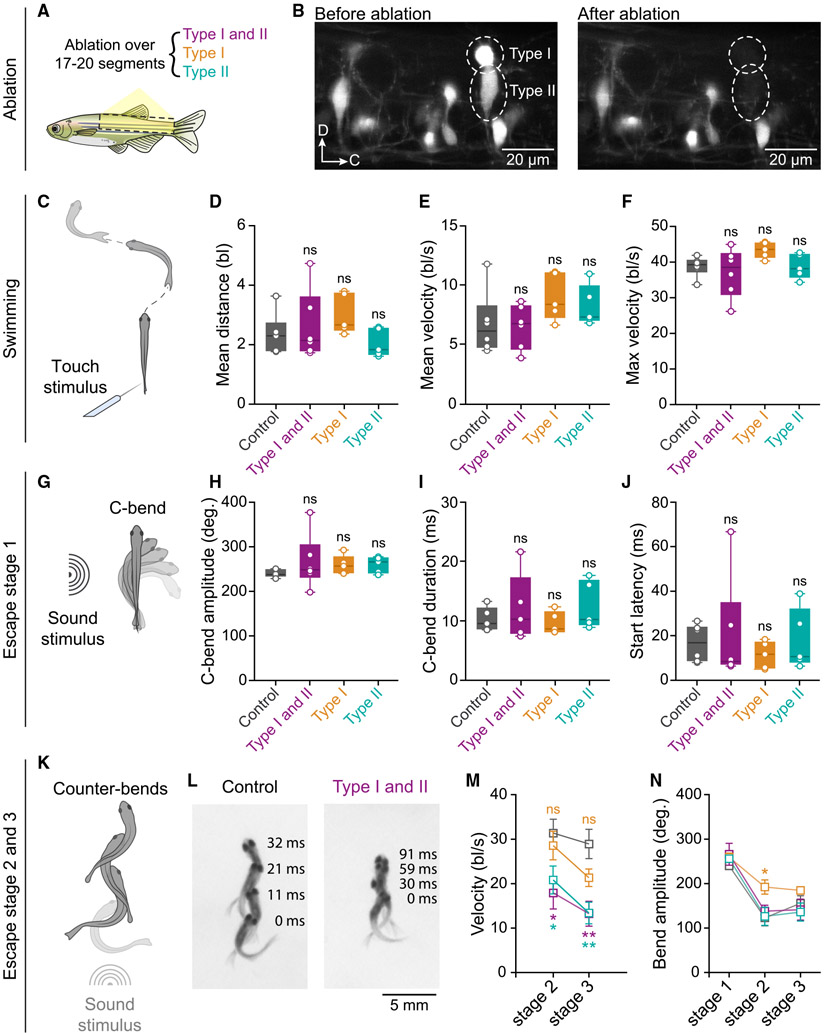
Effects of ablation of type I and II V2a INs on swimming and escape behavior (A) Ablation of V2a INs over the body region indicated in yellow and dashed lines. Three experimental groups were tested in which both type I and II were ablated (purple, n = 6 animals), or only type I (orange, n = 5 animals), or type II V2a INs (green, n = 5 animals, control in gray, n = 6 animals). (B) Image of the spinal cord before (left) and after (right) ablation of types I and II V2a INs encircled by the dotted lines. (C) Swimming was elicited by a touch stimulus. (D) Ablation of types I and II V2a INs had no effect on the swim distance. (E) Ablation of types I and II V2a INs had no effect on the swim velocity. (F) Ablation of types I and II V2a INs had no effect on the maximum velocity reached during swimming (in [D], [E], and [F] bl, body length; ns, not significant; one-way repeated-measures ANOVA). (G) Sound-induced escape behavior (C-bend, escape stage 1). (H) Ablation of types I and type II V2a INs had no effect on the C-bend amplitude. (I) Ablation of types I and type II V2a INs had no effect on the C-bend duration. (J) Ablation of types I and type II V2a INs had no effect on the C-bend latency (in [H], [I], and [J] statistical tests as in [F]). (K) Counter-bend and propulsion phase corresponding to escape stages 2 and 3. (L) Ablation of types I and II V2a INs decreased the distance traveled during escape. Superimposed images of the first four bends, time 0 ms corresponds to the C-bend. (M) Ablation of type II but not type I V2a INs alone decreased the velocity of escape stages 2 and 3 (data are presented as mean ± SEM, *p ≤ 0.05, **p ≤ 0.01; ns, not significant; two-tailed nonparametric Mann-Whitney test). (N) Effect of ablation of types I and II V2a INs on the bend amplitude during escape behavior (stages 1, 2, and 3, statistical test as in [M]).

The contribution of types I and II V2a INs to fast swimming and escape was probed *in vivo* using behavioral analysis combined with two-photon laser ablation. We performed selective laser ablation bilaterally over 17–20 spinal segments of both types I and II V2a INs, or only type I or type II V2a INs (Figures 7A and 7B; *n* = 6 animals with ablation of type I and II V2a INs, 5 animals with ablation of type I V2a INs, 5 animals with ablation of type II V2a INs, and 6 control animals). We first analyzed the effect of ablation of these V2a INs on fast swimming induced by touch of the tail skin. Ablation of both types of V2a INs or each type alone did not affect fast swimming, as there was no difference in the mean distance traveled, mean velocity, or maximum velocity compared with controls (Figures 7C-7F).

We then examined the effect of ablation of the two V2a IN types on sound-induced escape. The high-acceleration escape responses were described as occurring in three stages: stage 1, the “preparatory” stage, in which the body bends rapidly (C-bend), with minimal translation of the center of mass; stage 2, the “propulsive” stroke, when the fish accelerates away from its initial position; and stage 3, in which the fish either continues swimming or starts gliding (Domenici and Hale, 2019; Weihs, 1973). Ablation of types I and II V2a INs did not affect any of the evaluated parameters of escape stage 1 (C-bend; Figures 7G-7J). However, the subsequent escape stages 2 and 3 were significantly slower in animals in which both types I and II V2a INs were ablated (Figures 7K-7M), although their amplitude was similar to that of controls (Figure 7N). This resulted in a less effective escape with weaker propulsion and, hence, a shorter distance traveled during escape (Figure 7L). Selective ablation of type II V2a INs also significantly decreased the velocity of sound-induced escape stages 2 and 3 (Figure 7M) without affecting their amplitude (Figure 7N). In contrast, selective ablation of type I V2a INs alone did not significantly affect the velocity of escape stages 2 and 3 (Figure 7M), although a significant increase in the amplitude of stage 2 was seen in these ablated animals (Figure 7N).

Taken together, these results indicate that the early-established circuit between the two types of V2a INs and pMNs undergoes organizational and functional changes between larval and adult stages. While none of its components contribute to the generation of fast swimming, a subset of these neurons become incorporated within the escape circuit and contribute to propulsion efficiency.

## DISCUSSION

Spinal motor circuits are established early during development and enable animals to produce movements already at embryonic and neonatal stages (Branchereau et al., 2000; Clarac et al., 2004; Falgairolle et al., 2017; Hamburger 1963; Saint-Amant and Drapeau, 1998; Tong and McDearmid, 2012). During development and growth, all animals change size and shape. Many studies have shown changes in the activity of motor circuits during development (Combes et al., 2004; Hachoumi and Sillar, 2020; Marder and Rehm, 2005; Vinay et al., 2005), establishment of synaptic connections (Ha and Dougherty, 2018; Landmesser, 2018; Marin-Burgin et al., 2008), and modulation of the activity of the constituent neurons (Abbinanti et al., 2012; Husch et al., 2015; Smith and Brownstone, 2020). In this study, the experimental accessibility of zebrafish has allowed for a direct comparison of the connectivity and function of neuronal circuits at both larval and adult stages. Our previous work on the organization of the swimming network in adult zebrafish was exclusively limited to late-born ventrally located V2a INs and secondary MNs (Ampatzis et al., 2013, 2014; Ausborn et al., 2012; Gabriel et al., 2011; Song et al., 2018). Therefore, prior to the present study, no information had been available on the connectivity of the early-born V2a INs and pMNs in adult zebrafish. Our results now reveal that there is an age-dependent developmental reorganization of the circuit formed by the two early-born V2a IN types (type I and II) and pMNs in adult zebrafish compared to larval stages.

In larvae, types I and II V2a INs have been suggested to form a single interconnected circuit layer that controls the timing and amplitude of fast swimming activity (Menelaou and McLean, 2019). These two interneuron types make excitatory reciprocal connections with each other. In addition, type I V2a INs make broad and uniform connections with all pMNs innervating epaxial and hypaxial pMNs, but type II V2a INs instead connect only to epaxial or hypaxial pMNs (Bagnall and McLean, 2014; Menelaou and McLean, 2019). This V2a IN-pMN circuit is recruited only during fast swimming, but it has been shown that dopaminergic modulation can shift the recruitment of pMNs toward slower swimming frequencies (Jha and Thirumalai, 2020).

This early established circuit undergoes several key changes in connectivity and function during maturation. In adult zebrafish, the two V2a IN types do not form a reciprocal excitatory circuit; instead, there is only a unidirectional connection via chemical glutamatergic transmission from type I to type II V2a INs. These two V2a IN types are not recruited during swimming, and their ablation had no effect on swimming behavior *in vivo*. Ablation of type II V2a INs significantly impaired the efficiency of escape behavior in response to auditory stimuli, while type I V2a INs integrate sensory feedback. Furthermore, type I V2a INs lose their uniform connections with all pMNs in favor of strong and selective synaptic connections with the so-called caudal pMN, which innervates the ventralmost quadrant of the hypaxial axial musculature (Eisen et al., 1986; Menelaou and McLean, 2012; Westerfield et al., 1986). This synaptic connection is exclusively mediated via chemical transmission and shows a strong use-dependent facilitation and short-term potentiation during repetitive stimulation. The selective circuits in adult zebrafish formed by type I V2a INs and caudal hypaxial pMNs seem to be involved in sensory integration and could allow for steering during fast escape movements.

On the other hand, type II V2a INs establish new connections that enable them to provide broad and uniform excitation to all pMNs. These connections are mediated via mixed electrical and chemical synaptic transmission, which does not display any significant use-dependent augmentation or short-term plasticity. Type II V2a INs, in turn, receive a strong excitatory drive from the recently identified local cholinergic escape V2a INs in adult zebrafish (Guan et al., 2021). It is still unknown whether these cholinergic escape V2a INs exist in zebrafish larvae. In adults, these escape V2a INs act as a local relay of escape commands from the brain and control the onset and directionality of the C-bend (stage 1), while type II V2a INs may play a complementary role in propagating the local excitation through their widespread synaptic contacts with all pMNs throughout the whole spinal cord. Indeed, ablation of type II V2a INs did not affect the C-bend amplitude or duration in stage 1 of escape behavior but significantly decreased the velocity of the following counter-bends during stages 2 and 3 of escape. Our results show that the spinal motor network co-opts elements from swim-related larval circuits into a different escape-related circuit organization in adults. The broad excitatory drive to all pMNs from type II V2a INs could be related to the increase in body size that would require more efficient and powerful movements during escape.

Another major finding of this study is that the electrical coupling between V2a INs and pMNs, and the coupling among pMNs, play complementary functional roles. Gap junctions can act as low-pass filters that attenuate the transmission of spikes compared with slower membrane potential changes (Li et al., 2009). In addition, gap junctions allow changes in pMNs’ membrane potential to be propagated retrogradely to type II V2a IN presynaptic terminals where it modifies the probability of transmitter release and the resulting EPSPs in pMNs. Previously, we identified a similar retrograde influence between late-born secondary motoneurons and V2a INs driving swimming activity (Song et al., 2016). On the other hand, pMNs are strongly coupled to each other, and this mediates synchronization of their firing. Therefore, electrical coupling enables the pMNs to act as an ensemble that receives common excitatory drive from type II V2a INs and, once recruited, would synchronize their firing and at the same time, further strengthening their synaptic drive via retrograde influence onto type II V2a IN presynaptic terminals. This circuit construction with gap junctions both among postsynaptic pMNs and with presynaptic type II V2a INs would play a significant role in coordinating the synchronous activity of all pMNs to allow fast muscle contraction and generation of the force needed during escape behavior.

Robust behavioral output from birth to adulthood necessitates a continuous adaptation of the nervous system as new behaviors are acquired and as experience and interaction with the external environment adjusts the networks (Bucher et al., 2005; Dominici et al., 2011; Gerhard et al., 2017; Kämper and Murphey, 1994). Our understanding of the synaptic and circuit mechanisms underlying these adaptations is still incomplete. We show here that one of the earliest-established motor circuits undergoes major reconfiguration while zebrafish mature toward adulthood. Thus, the experimental accessibility of the same circuit components in zebrafish at different developmental stages has not only provided insights into the organization of motor circuits but is now starting to inform on the adaptive mechanisms during maturation. In conclusion, the circuit reorganization revealed in this study shows that during maturation there is a retention and redeployment of existing network components combined with a reconfiguration of their connectivity to suit the new demands associated with the growth and change in body shape as animals mature toward adulthood.

### Limitations of the study

There are specific limitations we wish to highlight. First, the distribution and location of synaptic contacts between the two types of V2a INs and pMNs require further analysis. Second, while the electrophysiological data show rectifying gap junctions between type II V2a INs pMNs, their molecular composition is still not fully resolved. This requires specific antibodies against other connexins that are not available. Third, we have tested the involvement of type I V2a INs in sensory integration by electrical stimulation of DRG neurons, but the identity of the sensory afferents and their role during a natural behavior remain to be clarified.

## STAR★METHODS

### RESOURCE AVAILABILITY

#### Lead contact

Further information and requests for resources and reagents should be directed to and will be fulfilled by the lead contact, Abdel El Manira (abdel.elmanira@ki.se).

#### Materials availability

This study did not generate new unique reagents.

#### Data and code availability

- All data reported in this paper will be shared by the lead contact upon request.
- This paper does not report original code.
- Any additional information required to reanalyze the data reported in this paper is available from the lead contact upon request.

### EXPERIMENTAL MODEL AND SUBJECT DETAILS

Zebrafish (*Danio rerio*) were raised and housed in the Karolinska Institutet, Comparative Medicine Biomedicum (KM-B) animal facility according to established procedures. Adult animals (8–11 week old; ~15 mm) of either sex were used for most experiments in this study, while larvae (4–5 dpf; ~4 mm) were only used for ablation of early-born neurons. The transgenic line Tg(*Chx10*:GFP), in which GFP expression in V2a INs is driven by the promoter of the transcription factor *Chx10*, was used for behavioral experiments, most electrophysiological recordings and anatomy experiments. In some experiments, the Tol-056 enhancer trap line (*Tol-056*), in which the Mauthner cells (M-cell) and cholinergic es-V2a INs express GFP, was crossed with Tg(*Chx10*-loxP-dsRed-loxP-GFP). The selective expression of channelrhodopsin 2 (ChR2) in glutamatergic neurons was obtained by crossing Tg(*vgluf2a*-loxP-dsRed-loxP-Gal4), Tg(UAS:ChR2-YFP), and Tg(*elavl3*:cre), referred to as Tg(vglut2-ChR2-YFP). All experimental procedures followed the EU guidelines and were approved by the Animal Research Ethical Committee in Stockholm.

### METHOD DETAILS

#### Ex-vivo adult zebrafish preparation

Electrophysiological recordings were performed using an *ex-vivo* preparation of adult zebrafish (8–11 week old) of either sex (Gabriel et al., 2008, 2011; Kyriakatos et al., 2011). Fish were deeply anesthetized with 0.03% Ethyl 3-aminobenzoate methanesulfonate (MS-222, Sigma-Aldrich, Cat# E10521) and then dissected in a slush of frozen extracellular solution containing (in mM): 134 NaCl, 2.9 KCl, 2.1 CaCl2, 1.2 MgCl2, 10 HEPES and 10 glucose, with pH of 7.8 adjusted with NaOH and osmolarity of 290 mOsm. The internal organs and axial musculature were removed, while the musculature of the tail was left intact, and an extracellular recording electrode was placed at an intramyotomal cleft to record ventral root motor activity. The vertebral arches were also removed to grant access to the spinal cord for whole-cell patch-clamp recordings. The skull was removed and in most experiments the brain was cut out leaving only the brainstem region. In some experiments only the telencephalon and the cerebellum were removed to allow access to the M-cells with a recording or a stimulation electrode. In experiments where a dorsal root ganglion was stimulated, two vertebral arches caudal to the intracellular recording site were kept intact while the axial musculature was carefully removed. The preparation was then placed in the recording chamber maintained at room temperature (20°C–22°C) and was continuously perfused with oxygenated extracellular solution for the duration of the experiment.

#### Electrophysiology

Neurons expressing fluorescent proteins (GFP, YFP or RFP) were visualized using a fluorescence microscope (Axioskop FS Plus, Zeiss) equipped with an IR-differential interference contrast (DIC) optics and a CCD camera with frame grabber (Hamamatsu). Type I and II V2a INs were distinguished by their soma position (type I V2a INs are the dorsal-most and medial-most V2a INs, while type II V2a INs are dorsal and located at the level of pMNs) and soma size (type II V2a INs have the largest soma size of all V2a INs). The intensity of GFP expression was also used to differentiate between type I and II V2 INs (type I V2a INs have higher GFP fluorescence than type II V2a INs). All recordings were performed using the whole-cell patch-clamp technique. Patch-clamp electrodes were pulled using a micropipette puller (P-1000, Sutter Instruments) from borosilicate glass (Hilgenberg) and were filled with an intracellular solution containing (in mM): 120 K-gluconate, 5 KCl, 10 HEPES, 4 Mg2ATP, 0.3 Na4GTP, 10 Na-phosphocreatine with pH 7.4 adjusted with KOH and an osmolality of 270–280 mOsm. In most experiments, 0.25% neurobiotin (Vector Laboratories Cat# SP-1120) was added to the intracellular solution to fill the neurons and allow for post-hoc morphology reconstruction. The electrodes resistance was 6–8 MΩ for pMNs and 8–12 MΩ for type I and II V2a INs. To access the neurons, holes were made with glass pipettes through the meninges into the spinal cord using motorized micromanipulators (SM7 Luigs & Neumann). New patch-clamp electrodes were then driven in from opposite directions while applying constant positive pressure and whole-cell patch-clamp recordings were performed from identified neurons. Sequential paired recordings were performed by keeping the recording of one of the neurons while extracting the other pipette and changing it to a clean one for the next recording. Intracellular signals were recorded in current-clamp with no bias current, or in voltage-clamp while holding the neuron at its resting membrane potential. The intracellular signal was amplified using a MultiClamp 700B amplifier (Molecular Devices) and low pass filtered at 10 KHz. Synaptic connectivity between pairs was tested by triggering a single action potential in the presynaptic neuron for 100–200 consecutive sweeps. To measure synaptic plasticity trains of ten action potentials were elicited in the presynaptic neuron (1 ms pulse duration, 40 Hz, 2 s sweep duration with no inter-sweep interval). Electrical coupling between pairs was tested by injection of hyperpolarizing current (1 s) in each neuron of the pair for 20–30 consecutive sweeps. In some experiments, the N-methyl-D-aspartate (NMDA) receptors antagonist D-(–)-2-amino-5-phosphonoic acid; (AP-5, 100 μM, Tocris, Cat# 0106/1), the a-amino-3-hydroxy-5-methyl-4-isoxazolepropionic acid (AMPA) receptor antagonist 2,3-dihydroxy-6-nitro-7-sulfamoyl-benzo[f]quinoxaline (NBQX, 50 μM, Tocris, Cat# 1044/1) and cadmium (Sigma-Aldrich, Cat# 202908) were bath applied to block the glutamatergic and all synaptic transmission, respectively. For dorsal root ganglia (DRG) stimulation, a fluorescent dye was injected into muscles that resulted in labeling of DRG sensory neurons. An extracellular stimulation electrode was placed on a DRG caudal to the whole-cell recording site. DRG neurons were stimulated with 1–2 ms current pulses every 3 s.

#### Fictive swimming and escape

Fictive locomotion was elicited by optogenetic activation of glutamatergic neurons in the hindbrain using the Tg(vglut2-ChR2-YFP) fish. Optogenetic stimulation was performed using a blue light source (CoolLED pE-4000, wavelength 460 nm) delivered for 10 to 50 s through a 63x objective (Zeiss) focused on the hindbrain. This reliably elicited swimming activity that lasted for the whole duration of the optogenetic stimulation. In these experiments, presumed type I and II V2 were targeted based on their location, soma size, and expression of YFP and were filled with neurobiotin for *post-hoc* morphological confirmation of their identity. Fictive escape was elicited by extracellular stimulation of the M-cell region (1 or 2 pulses stimulation, pulse duration 1 ms, 50 Hz). Fictive escape was first tested while recording a pMN to determine the minimum electrical stimulation amplitude that reliably elicited action potentials in the pMN characteristic of an escape response (Song et al., 2015). The same stimulation protocol was then used to elicit escape response while recording from type I or II V2a INs. In fictive swimming and fictive escape experiments, an extracellular suction electrode was placed on the tail musculature of the preparation to record peripheral nerve activity.

#### Neuronal tracing

Motoneurons and DRG neurons were back-labeled by injecting dextran dyes (Tetramethylrhodamine-dextran, MW 3000, Thermo Fisher, Cat# D3308; Alexa Fluor 647-dextran, MW 10000, Thermo Fisher Cat# D22914) into muscles. Crystals of the dyes were first dissolved in distilled water and subsequently desiccated on glass slides. Tg(*Chx10*:GFP) zebrafish were anaesthetized in 0.01% tricaine methanesulfonate (MS-222, Sigma-Aldrich) and placed lateral side up in a Petri dish. The tracers were injected with the tip of sharp tungsten pins in a selective quadrant of the fast axial musculature or in the full axial musculature (Figure S1). Animals were allowed to recover for at least 2 h prior to electrophysiology experiments. For post-hoc morphology analysis single V2a INs and pMNs were passively filled through the recording electrode with 0.25% neurobiotin. For dye-coupling experiments, one pMN was passively filled through the recording electrode with 2% neurobiotin.

#### Immunohistochemistry

Spinal cords with neurobiotin-filled neurons were dissected out and transferred into 4% paraformaldehyde (PFA) in phosphate buffer saline (PBS) (0.01 M; pH = 7.4) solution overnight at 4°C. The tissue was then washed three times for 5 min in PBS. Non-specific protein binding sites were blocked with 4% normal donkey serum and 1% bovine serum albumin (BSA; Sigma, Cat# A2153) and 0.5% Triton X-100 (Sigma, Cat# T9284) in PBS for 30 min at room temperature. Spinal cords were incubated with anti-GFP (chicken polyclonal, Abcam, Cat# ab13970) and streptavidin conjugated to either Alexa Fluor 647 (Invitrogen, Cat# S32357) or Alexa Fluor 405 (Invitrogen, Cat# S32351) to label neurobiotin-filled neurons. For connexins staining, we used rabbit anti-Cx35.5 (Fred Hutch Antibody Technology Facility, Miller laboratory, clone 12H5, 1:800) (Miller et al., 2017) and mouse IgG2A anti-Cx34.1 (Fred Hutch Antibody Technology Facility, Miller laboratory, clone 5C10A, 1:350) (Miller et al., 2017). After thorough buffer rinses the tissue was then incubated overnight at 4°C with the appropriate secondary antibody conjugated to Alexa Fluor–conjugated secondary antibody anti-chicken 488 (Invitrogen, Cat# A11039), anti-rabbit 568 (Invitrogen, Cat# A10042) or anti-mouse-647 (Invitrogen, Cat# A31571) (1:500 in 0.5% Triton X-100 in PBS). The spinal cords were then thoroughly rinsed in PBS and mounted in 80% glycerol in PBS.

#### Ablation and behavioral analysis

Tg(*Chx10*:GFP) zebrafish (6–7 week old) of either sex were anesthetized and embedded in 1.5% low-melt agarose in a Petri dish. The gills and mouth were exposed from the agarose and the Petri dish was filled with fish water containing 0.01% MS-222. They were then placed under a two-photon/confocal microscope (Zeiss LSM 980-Airy) and either type I or II V2a INs or both types were photoablated (wavelength 910 nm). Ablation of 20–30 V2a INs of each type was performed bilaterally over 17 to 20 segments. Control animals were embedded alongside the ablated zebrafish but were not subjected to two-photon laser ablation. Successful ablations were confirmed by the permanent loss of GFP fluorescence. Both the ablated and control zebrafish were allowed to recover from anesthesia and acclimate for 1 h before behavioral analysis at room temperature. Tg(*Chx10*:GFP) zebrafish larvae (4–5 dpf) were anesthetized in 0.005% MS-222 in fish water, then embedded in 1.5% low-melt agarose in a Petri dish and covered with fish water. A total of 10–20 type I or type II V2 INs were photo-ablated on each side of the spinal cord over 4–9 segments. These larvae were then raised until they reached 5 to 7 weeks of age and were used for morphological analysis.

For behavioral analysis, animals were placed in a circular dish containing fish water positioned on a plexiglass platform, illuminated from below by an LED lightbox and imaged from above with a high-speed camera. Control and ablated fish were tested in randomized order. Fish were placed in an 8 cm or 12 cm of diameter circular glass dish filled respectively with 25 or 50 mL of fish water and were allowed to acclimate for 20 min. Swimming was induced by a tactile stimulus applied to the tail using a fine tungsten pin and was recorded at 300 fps. Escape was evoked by a brief sound stimulus (10 ms, sine wave at 500 Hz) delivered by an audio speaker that was fixed on the plexiglass platform as previously described (Satou et al., 2009). Evoked escape was recorded at 470 fps. Trials in which stimulation failed to elicit a C-start escape maneuver were excluded from analysis.

#### Analysis of electrophysiological data

The criteria for including recorded neurons in this study were: a stable membrane potential at or below −50 mV, firing of action potentials at suprathreshold current injections, minimal changes in series resistance (<5%). The firing threshold was measured as the membrane potential at which the dV/dt exceeds 10 mV/s during the first elicited action potential. The input resistance was calculated as the slope of the linear part of the I-V curve obtained by injection of hyperpolarizing current steps. EPSP/Cs elicited by single action potentials were averaged over 50 to 200 consecutive sweeps and their amplitude was measured as the difference between baseline and EPSP/C peak. The amplitude of the EPSP/Cs elicited by high frequency trains of 10 action potentials was analyzed using a custom-made script in Matlab (MathWorks), which measures the amplitude of 10 individual EPSPs while subtracting the remaining depolarization from the preceding EPSPs by fitting their decay. The instantaneous swimming frequency was calculated using a custom-made script for Matlab as the inverse of the duration between the mid-point of consecutive swim bursts.

#### Image analysis and morphology reconstruction

Whole-mount imaging of the spinal cords was acquired using a laser scanning confocal microscope (Zeiss LSM 980-Airy or LSM 800). For overview images in Figures 1, 2, S1, and S2, consecutive orthogonal projections were stitched using the Photomerge function in Adobe Photoshop (Adobe Systems Inc., San Jose, CA). All figures and graphs were prepared with Adobe Photoshop and Adobe Illustrator (Adobe Systems Inc., San Jose, CA). The soma size was measured as the maximum surface area from confocal images. The axonal diameter was measured from confocal images one spinal segment away from the soma. The full morphologies (soma, axons, and dendrites) of neurobiotin-filled neurons were traced and reconstructed manually in Adobe Illustrator on z-stacks of confocal images.

#### Behavioral analysis

For each recording, the position of the centroid of the fish in each frame was extracted using the ImageJ (Schneider et al., 2012) plugin AnimalTracker (Gulyás et al., 2016). The full skeleton of the fish was tracked and the instantaneous velocity, distance traveled, and bend amplitudes were analyzed using a custom MATLAB script (Picton et al., 2021) with distance and velocity values expressed as body lengths (bl) and body lengths per second (bl/s), respectively. For fast swimming, several swimming episodes after a single touch stimulation were analysed, for a total of 10–60 swim cycles. Data are presented as averages of 4–6 recordings per animal.

### QUANTIFICATION AND STATISTICAL ANALYSIS

All statistical analysis was performed in Prism 7 (GraphPad software Inc.) and all data were tested for normality. Results were considered statistically significant if p < 0.05 (*p ≤ 0.05, **p ≤ 0.01, ***p ≤ 0.001, ****p ≤ 0.0001). A two-tailed Student’s t test (two groups), a one-way ANOVA with Tukey’s post hoc multiple comparisons (more than two groups, one condition) or a two-way ANOVA with Dunnett’s multiple comparisons (more than two groups, two conditions) were performed as appropriate. Data are reported as box and whiskers plot or as mean ± SEM with corresponding statistical tests and n numbers in figure legends.

## Supplementary Material

1

## Figures and Tables

**Table:** KEY RESOURCES TABLE

REAGENT or RESOURCE	SOURCE	IDENTIFIER
Antibodies		
Chicken polyclonal anti-GFP	Abcam	Cat# ab13970, RRID:AB_300798
Rabbit anti-Cx35.5 clone 12H5	Fred Hutch Antibody Technology Facility (Miller et al., 2017)	N/A
Mouse IgG2A anti-Cx34.1 clone 5C10A	Fred Hutch Antibody Technology Facility (Miller et al., 2017)	N/A
Secondary antibody anti-chicken Alexa Fluor 488	Invitrogen	Cat# A11039, RRID:AB_2534096
Secondary antibody anti-rabbit Alexa Fluor 568	Invitrogen	Cat# A10042, RRID:AB_2534017
Secondary antibody anti-mouse Alexa Fluor 647	Invitrogen	Cat# A31571, RRID:AB_162542
Streptavidin, Alexa Fluor 647 conjugate	Invitrogen	Cat# S32357
Streptavidin, Alexa Fluor 405 conjugate	Invitrogen	Cat# S32351
Chemicals, peptides, and recombinant proteins		
Ethyl 3-aminobenzoate methanesulfonate (MS-222)	Sigma-Aldrich	Cat# E10521
Neurobiotin	Vector Laboratories	Cat# SP-1120
PBS – Phosphate-Buffered Saline (10X) pH 7.4	Invitrogen	Cat# AM9625
Triton X-100	Sigma-Aldrich	Cat# T9284
Bovine Serum Albumin	Sigma-Aldrich	Cat# A2153
Tetramethylrhodamine-dextran	Thermo Fisher	Cat# D3308
Alexa Fluor 647-dextran	Thermo Fisher	Cat# D22914
D-(-)-2-amino-5-phosphonoic acid (AP-5)	Tocris	Cat# 0106/1
2,3-dihydroxy-6-nitro-7-sulfamoyl-benzo[f] quinoxaline (NBQX)	Tocris	Cat# 1044/1
Cadmium	Sigma-Aldrich	Cat# 202908
Experimental models: Organisms/strains		
*Danio rerio*: Tg(*Chx10*:GFP)	(Kimura et al., 2006)	ZFIN: ZDB-GENO-010924-10
*Danio rerio:* Tg(*Chx10*-loxP-dsRed-loxP-GFP)	(Kimura et al., 2006)	ZFIN: ZDB-TGCONSTRCT-070117-143
*Danio rerio*: Tol-056:GFP	(Satou et al., 2009)	ZFIN: ZDB-FISH-150901-13314
*Danio rerio*: Tg(*VGlut2a*-loxP-dsRed-loxP-Gal4)	(Satou et al., 2013)	ZFIN: ZDB-TGCONSTRCT-131127-2
*Danio rerio*: Tg(*elavl3*:cre)	(Förster et al., 2017)	ZFIN: ZDB-TGCONSTRCT-170921-3
*Danio rerio*: Tg(UAS:ChR2-YFP)	(Fidelin et al., 2015)	ZFIN: ZDB-TGCONSTRCT-150324-2
Software and algorithms		
pClamp	Molecular Devices	RRID: SCR_011323; https://www.moleculardevices.com/
Spike2	Cambridge Electronic Design	RRID: SCR_000903; https://ced.co.uk
MATLAB	The MathWorks, Inc., Natick, Massachusetts, United States.	RRID: SCR_001622; https://www.mathworks.com
ImageJ	(Schneider et al., 2012)	RRID: SCR_003070; https://imagej.nih.gov/ij/
Adobe Photoshop	Adobe Systems Inc., San Jose, CA	RRID:SCR_014199; https://www.adobe.com/products/photoshop.html
Adobe Illustrator	Adobe Systems Inc., San Jose, CA	RRID:SCR_010279; http://www.adobe.com/products/illustrator.html
ZEN Digital Imaging for Light Microscopy	Carl Zeiss Microscopy GmbH, Jena, Germany	RRID:SCR_013672; http://www.zeiss.com/microscopy/en_us/products/microscope-software/zen.html#introduction
Hiris v.5.2.0	R&D Vision	N/A
GraphPad Prism7	GraphPad Software	RRID: SCR_002798; https://www.graphpad.com

## Highlights

- Components of an early-established motor circuit are maintained in adult zebrafish.
- Their connectivity undergoes major reconfiguration during maturation.
- During maturation, circuit components are redeployed to a different motor function.
