# Supplementary material for: Transformation of an early-established motor circuit during maturation in zebrafish
Source: Cell Rep. Author manuscript; Available in PMC 2022 May 5. (PMC9071512; doi:10.1016/j.celrep.2022.110654)
Supplement: 1 [file NIHMS1798482-supplement-1.pdf]

Cell Reports, Volume *39*

## Supplemental information

# Transformation of an early-established motor circuit during maturation in zebrafish

**Irene Pallucchi, Maria Bertuzzi, Jennifer Carlisle Michel, Adam C. Miller, and Abdeljabbar El Manira**

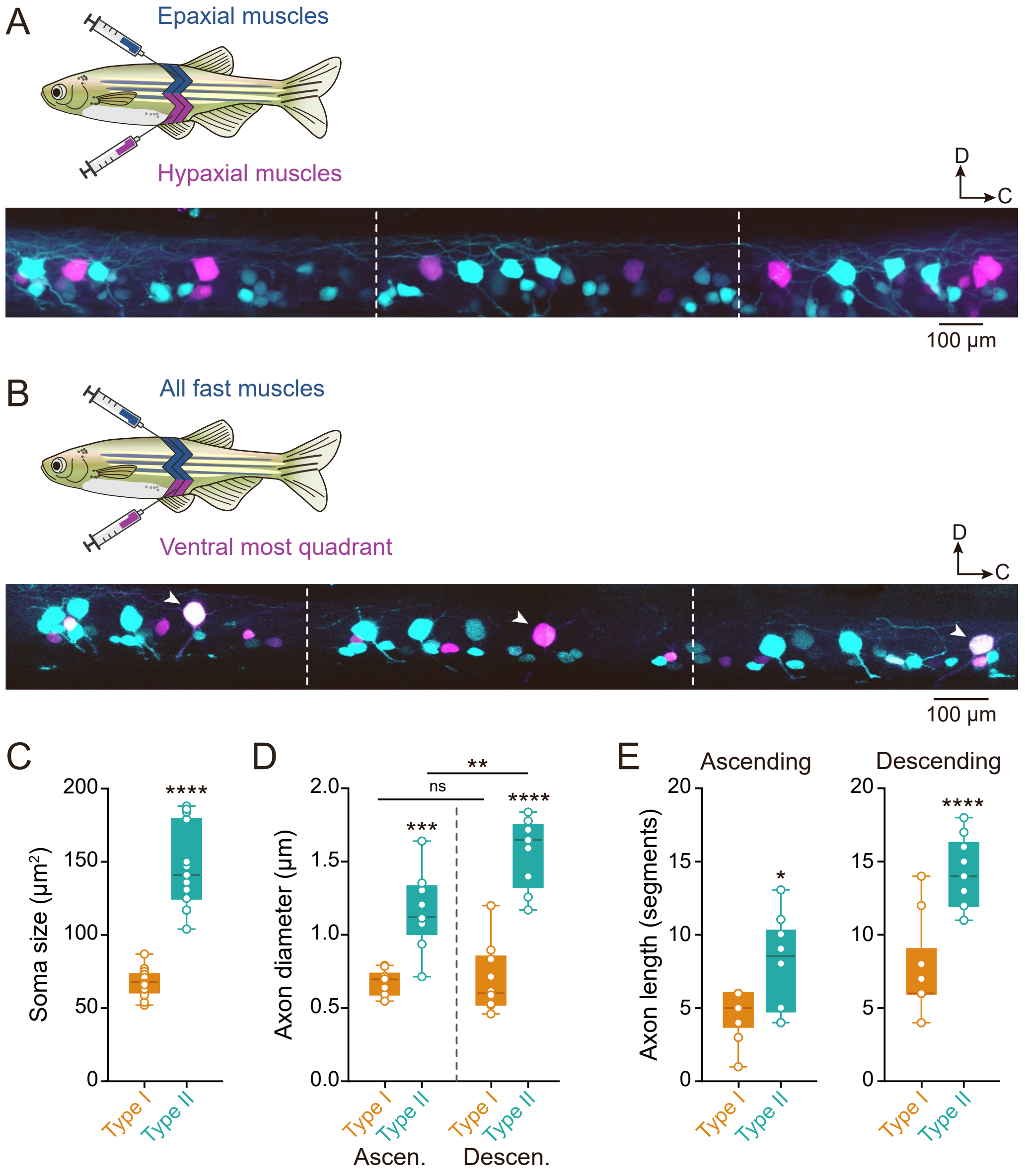


**Figure S1. pMNs innervate different quadrants of the axial musculature. Related to Figure 1.**
(A) Top: injection of retrograde dextran dyes in epaxial and hypaxial muscles in intact zebrafish. Bottom: images of three spinal hemisegments showing the position of pMNs innervating epaxial (cyan) and hypaxial (magenta) muscles. Dashed lines indicate segment borders ($n$ = 85 segments, 11 animals). (B) The ventral most quadrant of axial musculature (magenta) was injected with a retrograde dextran dye while a different dextran dye was injected in all axial musculature (cyan). The caudal most pMN in each spinal hemisegment always innervates the ventral most muscle quadrant (arrowhead, $n$ = 57 segments, 5 animals). (C) Soma size of type I and II V2a INs. (****p≤ 0.0001, unpaired two tailed Student's t-test, $n$ = 15 type I V2a INs and 15 type II V2a INs). (D) Diameter of type I and II V2a IN ascending (Ascen.) and descending (Descen.) axonal projections (ns: not significant, **p≤0.01, ***p≤0.001, ****p≤0.0001, one-way ANOVA with Tukey's multiple comparisons, $n$ = 10 type I V2a INs and 9 type II V2a INs). (E) Length of type I and II V2a IN ascending and descending axonal projections (ns: not significant, *p≤0.05, ***p≤0.001, ****p≤0.0001, two-tailed unpaired student's t-test, $n$ = 10 type I V2a INs and 10 type II V2a INs).

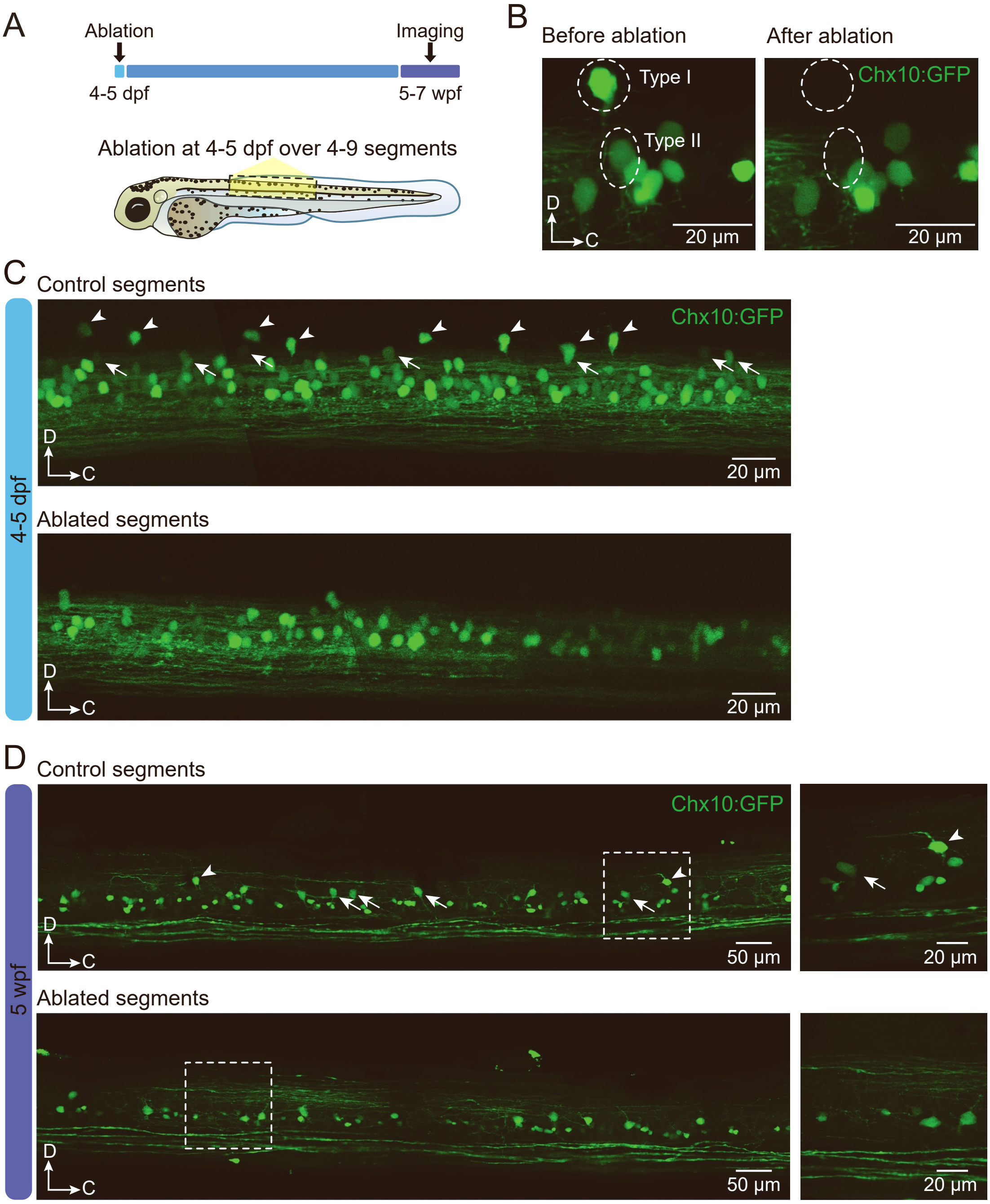


**Figure S2. Confirmation of the identity of type I and II V2a INs using chronic ablation. Related to Figure 1.** (A) Timeline of the experiments with chronic ablation of type I and II V2a INs in the mid-body region in zebrafish larvae (dpf, days post fertilization; wpf, weeks post fertilization, $n = 6$ animals) (B) Images showing type I and II V2a INs in larval zebrafish before and after ablation. (C) Images of larval zebrafish spinal cord showing control segments with intact type I and II V2a INs (top), and segments in which these two V2a IN types were ablated (bottom). Arrowheads and arrows indicate type I and II V2a INs, respectively. (D) Right: images of juvenile/adult zebrafish spinal cord showing two segments with intact type I and II V2a INs (top), and segments in which both V2a IN types were ablated. Arrowheads and arrows indicate type I and II V2a INs, respectively. Left: expansion of areas indicated by dashed boxes.

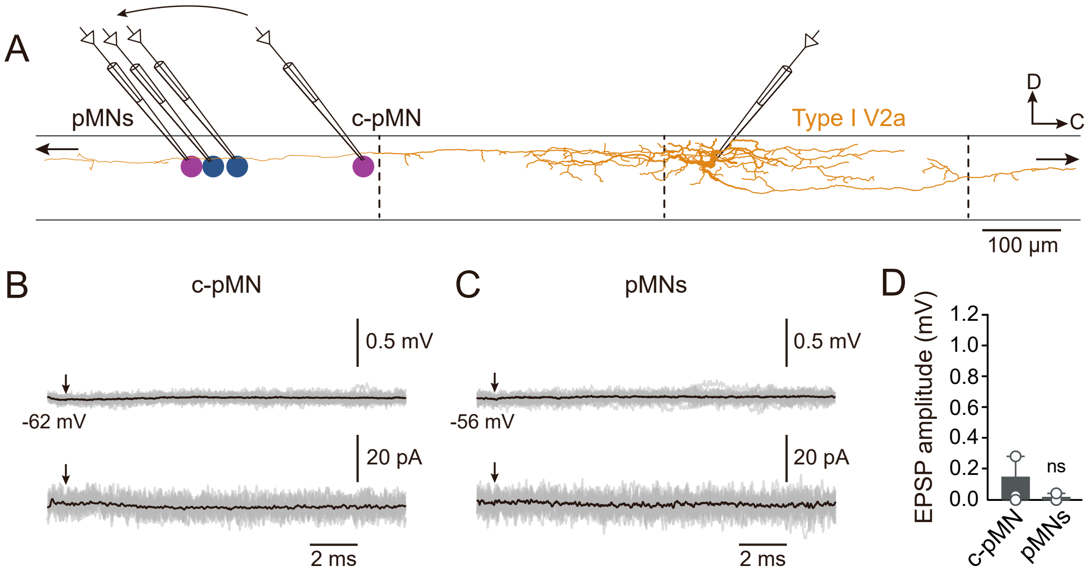


**Figure S3. Type I V2a INs do not connect to pMNs in rostral segments. Related to Figure 3.**
(A) Schematic of the experimental setup for sequential paired recording. (B-C) Type I V2a IN stimulation did not elicit any EPSP/C neither in the caudal pMN (c-pMN) nor in other pMNs. Grey traces: 20 individual sweeps, black traces: averages of 150 sweeps. Arrows indicate V2a IN action potentials. ($n$ = 5 c-pMN, $n$ = 3 other pMNs, 6 animals). (D) Quantification of the EPSP amplitude in the c-pMNs and other pMNs located in segments rostral to the V2a IN (ns: not significant, two-tailed unpaired student's t-test).

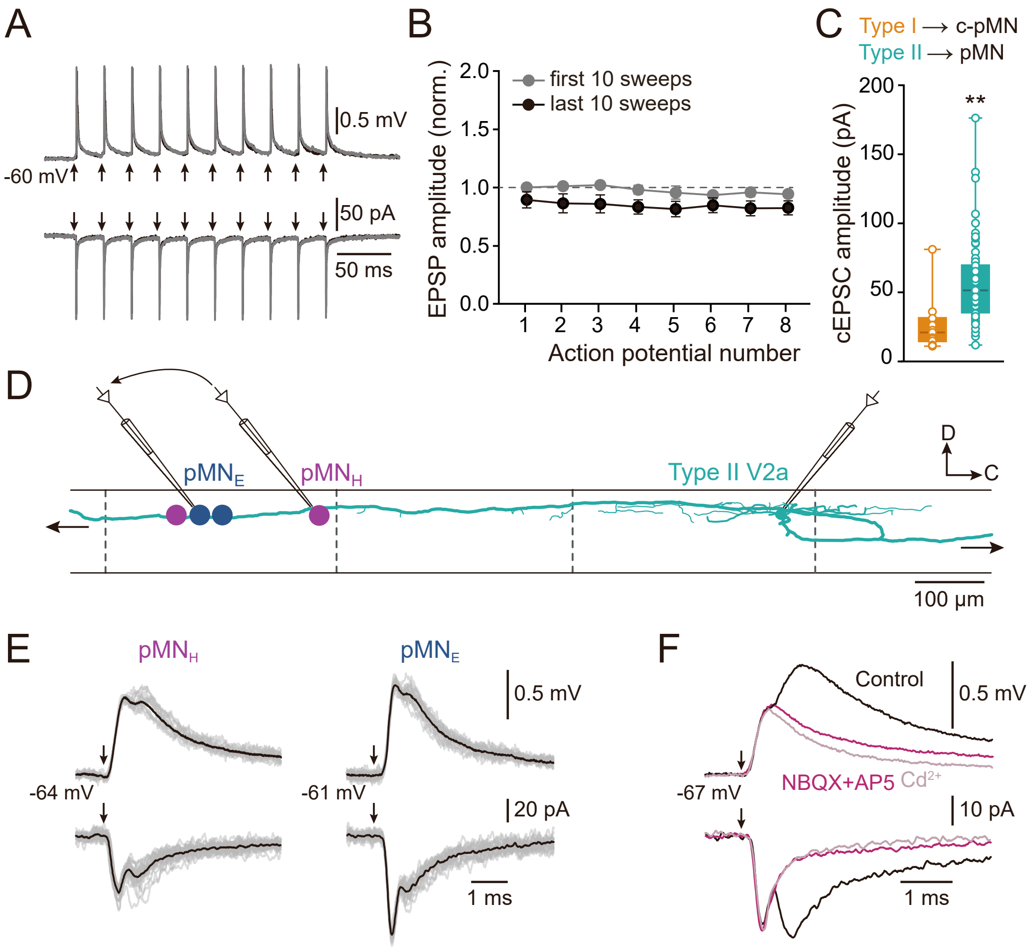


**Figure S4. Lack of short-term facilitation at synapses between type II V2a INs and pMNs. Related to Figure 4.**

(A) Example recording from a pMN showing a lack of short-term facilitation of EPSPs induced by 40 Hz stimulation of a type II V2a IN (*n* = 6 pairs, 6 animals). (B) Quantification of EPSP amplitude induced in pMNs in response to 40 Hz stimulation of type II V2a INs (data are presented as mean ± SEM, one-way and two-ways repeated measures ANOVA, data were not significantly different). (C) Amplitude of EPSCs induced in pMNs by stimulation of type I (orange) and type II (green) V2a INs (***p≤0.001, two-tailed unaired student's t-test, *n* = 13 type I, 63 type II V2a INs). (D) Experimental setup for sequential paired recording. A type II V2a IN was stimulated while epaxial (pMN$_E$) or hypaxial (pMN$_H$) pMNs located two segments rostrally were recorded sequentially (*n* = 19 V2a INs, 18 animals). (E) Example traces of EPSP/Cs in a pMN$_H$ (left) or a pMN$_E$ (right) induced by stimulation of the same type II V2a IN. Grey traces: 20 individual sweeps, black, pink and magenta traces: averages of 50 sweeps. Arrows indicate V2a IN action potentials. (F) Type II V2a IN stimulation produced a mixed chemical and electrical EPSP/Cs in a rostral pMN. The chemical EPSP was abolished by NBQX and AP5 leaving an electrical EPSP that remained unchanged also in presence of cadmium ($Cd^{2+}$).

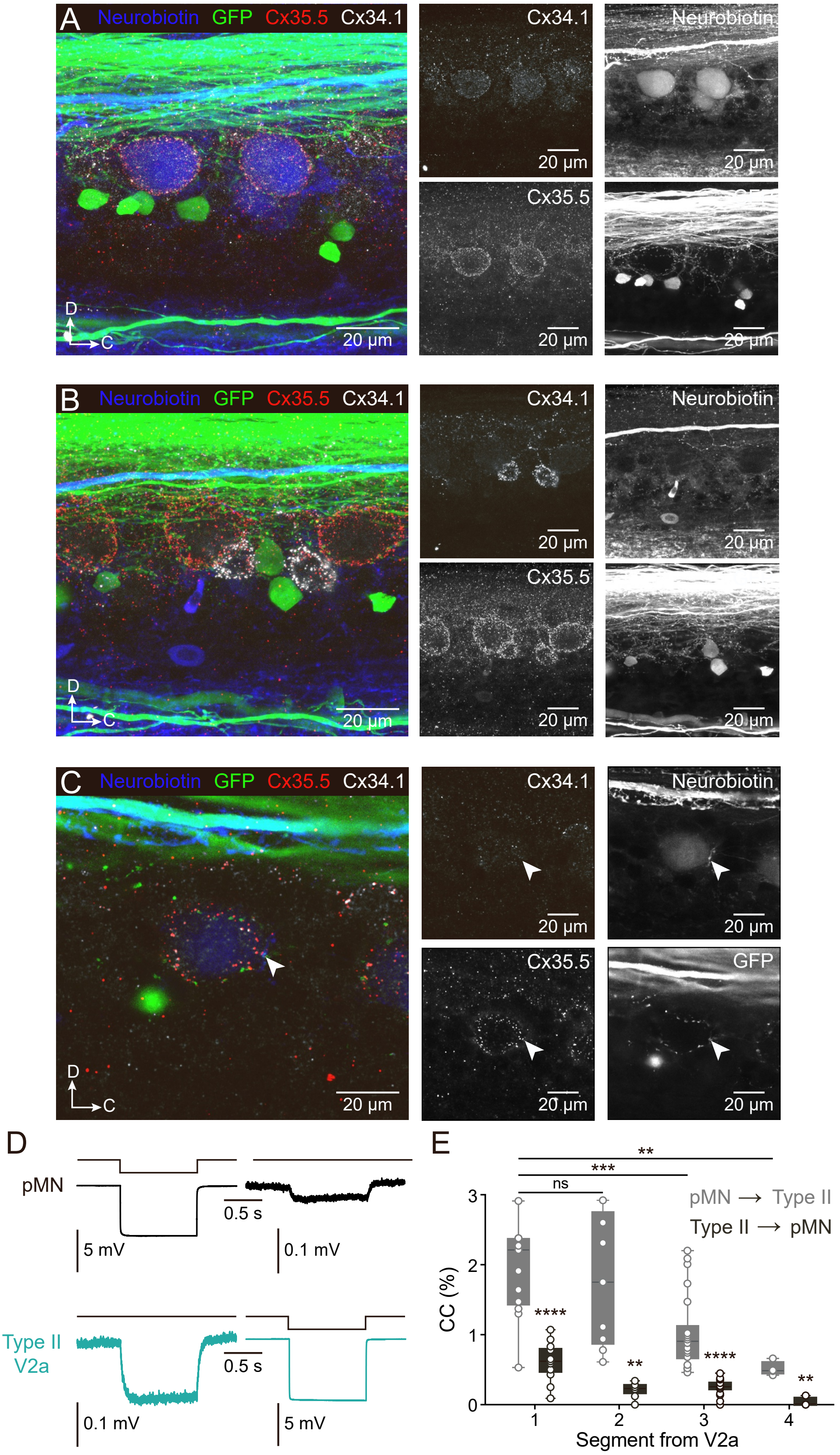


**Figure S5. Connexin composition of gap junctions between type II V2a INs and pMNs. Related to Figure 5.** (A) Image of neurobiotin-filled pMNs decorated with connexin (Cx) 35.5, while Cx34.1 immunoreactivity was sparse or absent in these pMNs. (*n* = 6 animals). (B) Cx34.1 puncta were abundant in other unidentified neurons in the same preparation. (C) High resolution image showing Cx35.5 puncta on a pMN. Cx35.5 puncta were adjacent but did not colocalize with axon collaterals (arrowhead) of type II V2a INs. (D) Representative recordings of a type II V2a IN and a pMN located three segments apart showing that they are electrically coupled. Traces are averages of 30 sweeps. (*n* = 48 pairs, 25 animals) (E) Plot of the coupling coefficient between pMNs and type II V2a INs located at different spinal segments. (*$p \leq 0.05$, **$p \leq 0.01$, ***$p \leq 0.001$, ****$p \leq 0.0001$, one-way repeated measures ANOVA corrected with Dunnett's multiple comparison test).

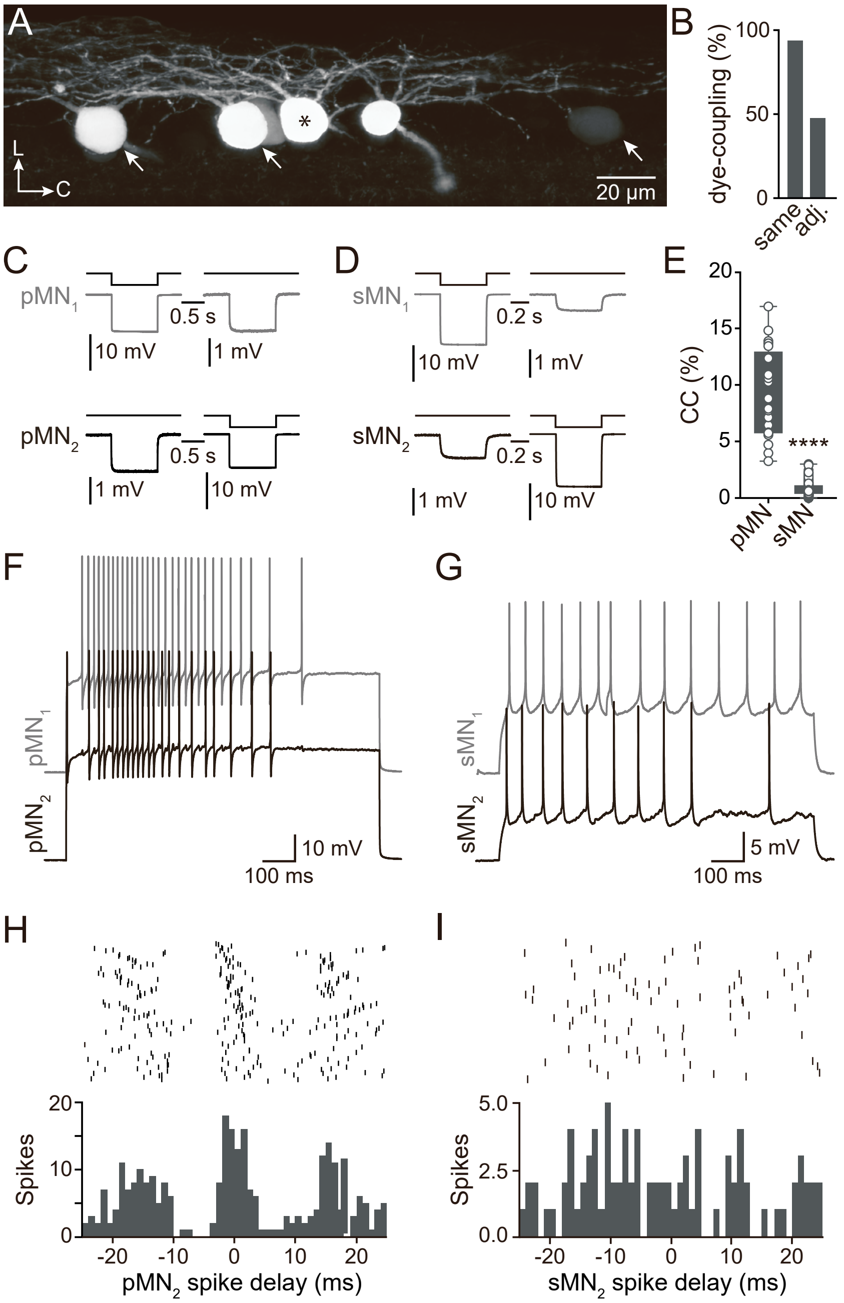


**Figure S6. Gap junctions between pMNs promote their firing synchrony. Related to Figure 5.**
(A) Neurobiotin injection into a pMN (asterisk) resulted in dye-coupling in all other pMNs (arrows) in the same hemisegment (*n* = 5 filled pMNs, 4 animals). (B) Percentage of dye-coupled pMNs in the same segment (same) and in adjacent segments (adj.). (C) Representative recordings showing bidirectional electrical coupling between a pair of pMNs located in the same segment (*n* = 14 pairs, 7 animals). (D) Representative recordings showing bidirectional electrical coupling between a pair of sMNs located in the same segment (*n* = 56 pairs, 35 animals). (E) Coupling coefficient between pairs of sMNs or pMNs (****$p \leq 0.0001$, unpaired two tailed Student's t-test). (F) Example recordings of a pair of electrically coupled pMNs firing in synchrony (*n* = 6 pairs, 4 animals). (G) Example recordings of a pair of electrically coupled sMNs with asynchronous firing (*n* = 3 pairs, 2 animals). (H) Quantification of the spike timing in $pMN_2$ relative that of $pMN_1$ showing that the two pMNs fire synchronously (same pMN pair as in F). (I) Quantification of spike timing between the pair of sMNs as in (G) showing the lack of synchronous firing of the two sMNs.

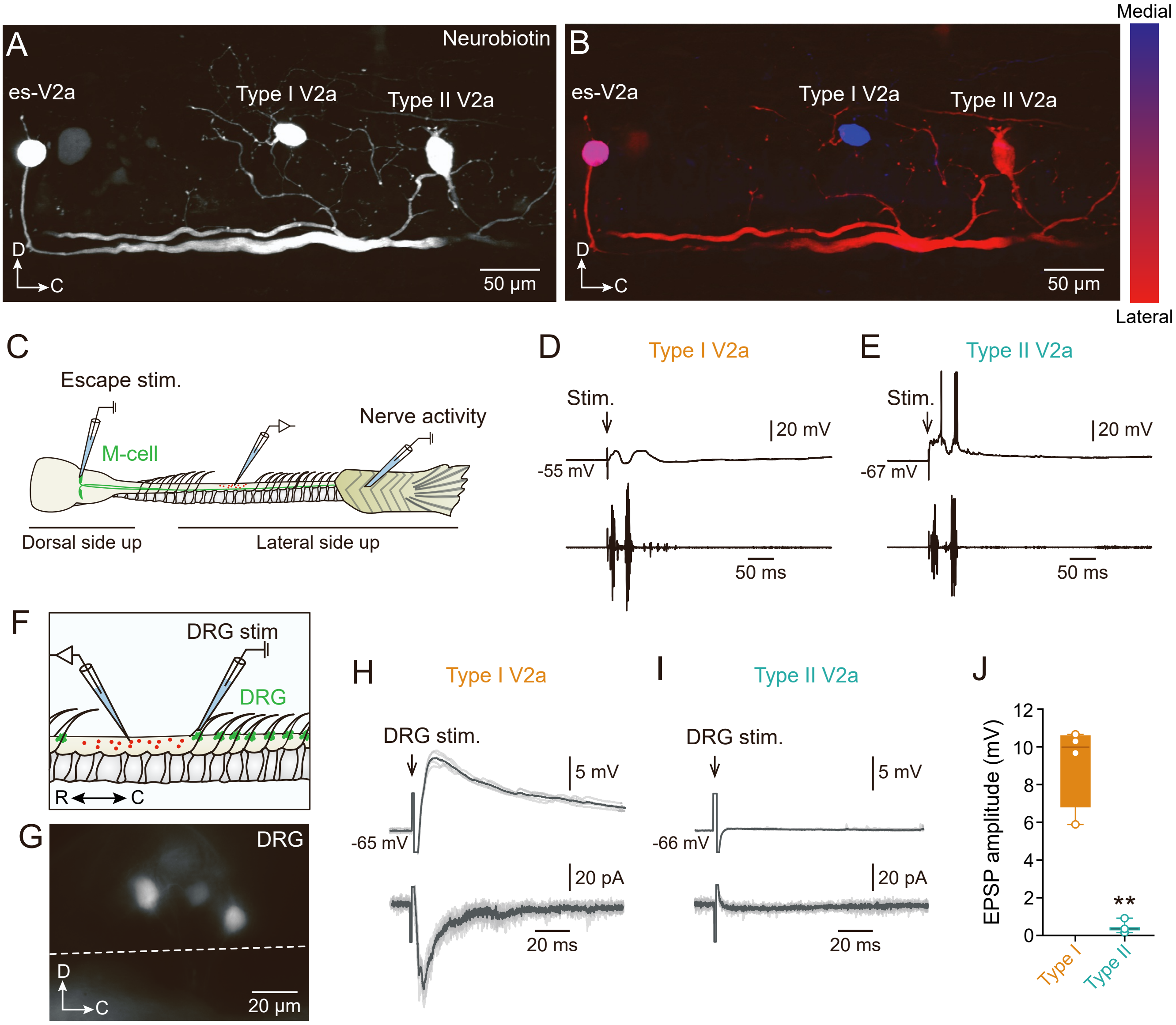


**Figure S7. Type I and II V2a INs are embedded in different functional circuits. Related to Figure 6.** (A) Image showing a type I, a type II and an es-V2a IN. (B) Depth analysis showing the mediolateral location of the three V2a IN types in the medial spinal cord. (C) Experimental setup for fictive escape. An extracellular stimulating electrode was placed above the M-cell to elicit escape while recording from a type I or II V2a IN along with extracellular recording of the motor activity in a peripheral nerve. (D) Example recording showing a type I V2a IN that was not recruited during escape activity induced by stimulation of M-cell region ($n$ = 5 type I, 3 animals). (E) Example recording of a type II V2a IN that was reliably recruited during escape induced by stimulation of M-cell area ($n$ = 5 type II, 3 animals). (F) Experimental setup used for stimulation of DRG neurons. (G) Image showing back-labeled DRG neurons by injection of a dye into muscles. Dashed line indicates the dorsal margin of the spinal cord. (H) Example EPSP/Cs induced in a type I V2a IN by stimulation of DRG neurons. Grey traces: 5 individual sweeps, black traces: averages of 10 sweeps ($n$ = 4 type I, 3 animals). (I) Example recording of a type II V2a IN that did not receive any EPSP/Cs from DRG neurons (n = 3 type II, 3 animals). (J) EPSP amplitude in type I (orange) and II (green) V2a INs in response to DRG stimulation.
